# Immunoinformatic analysis of the whole proteome for vaccine design: An application to *Clostridium perfringens*

**DOI:** 10.3389/fimmu.2022.942907

**Published:** 2022-08-30

**Authors:** Luis F. Soto, Ana C. Romaní, Gabriel Jiménez-Avalos, Yshoner Silva, Carla M. Ordinola-Ramirez, Rainer M. Lopez Lapa, David Requena

**Affiliations:** ^1^ Escuela Profesional de Genética y Biotecnología, Facultad de Ciencias Biológicas, Universidad Nacional Mayor de San Marcos, Lima, Peru; ^2^ Departamento de Ciencias Celulares y Moleculares, Laboratorio de Bioinformática, Biología Molecular y Desarrollos Tecnológicos, Facultad de Ciencias y Filosofía, Universidad Peruana Cayetano Heredia (UPCH), Lima, Peru; ^3^ Departamento de Salud Pública, Facultad de Ciencias de la Salud, Universidad Nacional Toribio Rodríguez de Mendoza de Amazonas, Chachapoyas, Peru; ^4^ Instituto de Ganadería y Biotecnología, Universidad Nacional Toribio Rodríguez de Mendoza de Amazonas, Chachapoyas, Peru; ^5^ Laboratory of Cellular Biophysics, The Rockefeller University, New York, NY, United States

**Keywords:** immunoinformatics, Clostridium perfringens, epitope, toxin, vaccine, molecular dynamics

## Abstract

*Clostridium perfringens* is a dangerous bacterium and known biological warfare weapon associated with several diseases, whose lethal toxins can produce necrosis in humans. However, there is no safe and fully effective vaccine against *C. perfringens* for humans yet. To address this problem, we computationally screened its whole proteome, identifying highly immunogenic proteins, domains, and epitopes. First, we identified that the proteins with the highest epitope density are Collagenase A, Exo-alpha-sialidase, alpha n-acetylglucosaminidase and hyaluronoglucosaminidase, representing potential recombinant vaccine candidates. Second, we further explored the toxins, finding that the non-toxic domain of Perfringolysin O is enriched in CTL and HTL epitopes. This domain could be used as a potential sub-unit vaccine to combat gas gangrene. And third, we designed a multi-epitope protein containing 24 HTL-epitopes and 34 CTL-epitopes from extracellular regions of transmembrane proteins. Also, we analyzed the structural properties of this novel protein using molecular dynamics. Altogether, we are presenting a thorough immunoinformatic exploration of the whole proteome of *C. perfringens*, as well as promising whole-protein, domain-based and multi-epitope vaccine candidates. These can be evaluated in preclinical trials to assess their immunogenicity and protection against *C. perfringens* infection.

## Introduction

*Clostridium perfringens* is a Gram-positive bacterium frequently associated with systemic and enteric diseases (1). In humans, *C. perfringens* is one of the most common food-poisoning causing bacteria, responsible for 1,000,000 cases per year in the US (2). Over a thousand cases result in gas gangrene, which can take from hours to weeks to develop depending on the tissue oxygen levels (3). It has a 10-30% mortality rate when treated, but 100% when untreated (4). Moreover, it is a pathogen known as a biological warfare weapon (5, 6). Therefore, it is necessary to develop preventive tools, like the identification of proteins and domains that can be used for molecular diagnostics and vaccines.

The histotoxic infections caused by this bacterium include gas gangrene in contaminated wounds, and several symptoms of human gastrointestinal diseases by either food- or non-food-borne *C. perfringens* infection (7, 8). *C. perfringens* isolates are classified into five toxinotypes, based on the production of four major toxins: α, β, ϵ and ι (1). *C. perfringens* type A is the main toxinotype that infects humans, producing gas gangrene, food poisoning, and non-foodborne gastrointestinal disease (9). Its main mechanism of cell invasion depends on the formation of a pore in the host cell membrane. The phospholipase C (cpa) and perfringolysin O (pfo) are involved in histotoxic infections, while the enterotoxin (etx), the *β* toxin (cpb) and the *β*-like toxin, the epsilon toxin (cpe) are involved in intestinal diseases (9, 10).

When *C. perfringens* toxins enter host cells, they are cut into small peptides by the proteasomes (11). Similarly, the whole bacterium can also be phagocytized and degraded by the endolysosomes into peptides. In both scenarios, the HLA (human leukocyte antigen) molecules (class I and class II, respectively) bind to these peptides and display them on the cell surface. Then, the HLA-peptide complexes are recognized by the TCR receptor in the surface of CD8+ and CD4+ immature T-cells, respectively, triggering an adaptive immune response. They will mature into cytotoxic T (CTL) and T-helper (HTL) lymphocytes. CTLs will produce a cytotoxic response against the infected cells, whereas HTL will stimulate the proliferation of antigen-specific B cells by clonal expansion, generating specific antibodies against the pathogen (12).

Vaccination is the most cost-effective method to prevent diseases (13). Although there is a vaccine available against *C. perfringens* for sheep and goats (14), there is no one approved for humans (5). Traditional vaccine development approaches are based on whole attenuated or dead microorganisms, and inactivated bacterial toxins. Nevertheless, they present the risk of potential reactivation or recombination of the vaccine strain, as well as offering limited protective effectiveness and immunity versus newer technologies (13). An alternative is subunit-based vaccines, consisting in one or more domains of antigenic proteins. Generally, these protein domains should be easily accessible, such as the external region of membrane proteins. As example, the NVX-CoV2373, a protein subunit-based vaccine producing the recombinant Spike protein, have demonstrated to neutralize the virus in different organism models and in humans (15, 16).

A new kind of vaccine, called the multi-epitope vaccine, has gained popularity in recent years (17). It consists of a novel protein connecting immunogenic epitopes. This kind of vaccine provides certain advantages over classical vaccines and single-epitope vaccines, such as a broader spectrum of pathogen variants, an optimized design that elicits both CTL, HTL, and B cell responses, and reduced adverse effects (18). It relies on the identification of epitopes through computational prediction and experimental testing. Epitopes are selected based on different criteria and assembled into a construct for their delivery to the immune system machinery. Multi-epitopes vaccines have already shown good results when tested in human clinical trials. For example, EMD640744 and Reniale have shown immunologic efficacy against advanced solid tumors (19) and in reducing tumor progression (20), respectively. Also, multi-epitope vaccines developed to trigger cross-immunity against different strains of influenza have shown great immunogenicity (21). Additionally, in mice, a multi-epitope vaccine has shown protective immunity against Toxoplasma gondii (22).

Nowadays, immunoinformatics tools help to massively screen protein sequences. They allow the computational identification of antigenic proteins and epitopes, reducing development time and cost (23). They rely on experimental data, which is available in databases like the Immune Epitope Database (IEDB) (24). It collects data of antibodies and CTL-, HTL- and B-epitopes, detected or evaluated in humans and other animal species (24, 25). Furthermore, there are predictors of CTL- and HTL-epitopes based on artificial intelligence tools and trained on experimental information. Among them, we have NetMHCpan I and II (26, 27) and MHCFlurry (28), which are currently the best according to independent evaluations (29). Additionally, Epitope-Evaluator performs comparative analysis of the outputs of these predictors, allowing an easy and graphical identification of highly antigenic proteins, as well as conserved promiscuous epitopes (30). Protein modeling and molecular dynamics (MD) allow studying structural characteristics of antigenic proteins, such as flexibility, disorder degree, and solvent accessibility, among others. These features are related to the immune response elicited (31–34). This could be because flexible and disordered antigens have more different conformations available at the moment of binding to the immune system molecules, maximizing favorable interactions (33).

In the present study, we predicted immunogenic epitopes from the 2721 proteins that comprise the known proteome of *C. perfringens* type A, to identify vaccine candidates following 3 different approaches. First, we used epitope prediction to identify the proteins containing the highest number of epitopes that may elicit a good immunogenic response when used as recombinant vaccines. Second, we analyzed only the toxins and determined which non-toxic regions of these proteins are rich in HTL-epitopes and could be used as a vaccine, without the histotoxic damage. Third, using the best candidate epitopes, we generated synthetic constructs and studied their structural characteristics such as the flexibility and the accessibility of the epitopes through molecular dynamics. As result, we are presenting novel candidates for further testing as potential vaccines.

## Material and methods

### Data retrieval

#### Protein sequences and selection

**Figure 1:**
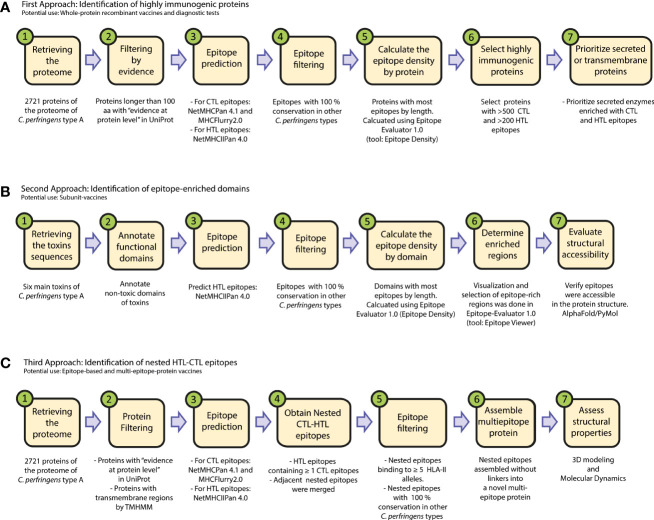
Flowchart of the immunoinformatic exploration of pathogens for vaccine development. We present three workflows to identify: **(A)** immunogenic proteins for protein-based vaccines, **(B)** protein domains enriched in HTL epitopes for subunit vaccines, and **(C)** nested epitopes for the design of novel multi-epitope protein vaccines. The proteins obtained in these three approaches might be used as well in immunodiagnostic tests.

We downloaded amino acid sequences of the 2721 proteins reported for *C. perfringens* Type A in the UniProt database, using the reference proteome with ID:UP000000818 (**Supplementary Table 1**). We explored the proteome, following three approaches (**Figure 1**).

In the first approach, we sought to identify proteins with most epitopes. So, the “epitope density” of each protein was calculated, which was defined as the number of epitopes of the protein divided by the length (in aa) of the protein. This was calculated using the tool “Epitope Density” from Epitope-Evaluator (https://fuxmanlab.shinyapps.io/Epitope-Evaluator/) (30). Only proteins longer than 100aa with “evidence at protein level” according to the UniProt database were considered in this approach (**Figure 1A**).

In the second approach, the epitopes within toxins were analyzed, using the following protein sequences: perfringolysin O (P0C2E9), enterotoxin A (Q8XKY4), enterotoxin B (Q8XKP0), enterotoxin D (Q8XMT2), beta2-toxin (Q93MD0) and phospholipase C (P0C216), as previously reported (10, 35). To propose a subunit-based vaccine that induces an appropriate humoral response, we further studied the HTL-epitopes in the non-toxic domains of these proteins. Among them, only the non-toxic domain of Perfringolysin O is well characterized, so we further studied this region. This protein is one of the most immunogenic toxins of *C. perfringens* (35), and its non-toxic domain is between the amino acid position 1 and 363, while the toxic and binding domains are reported to be between 363 and 472 (35, 36). This analysis was performed using the “Epitope Location” tool of Epitope-Evaluator (30) and the tridimensional model obtained by AlphaFold2 (**Figure 1B**).

In the third approach, we started with all the epitopes in the proteins of *C. perfringens* Type A and applied consecutive filters, as described below (see **Figure 1C**).

#### Selection of HLA class-I and -II alleles

Each population has a different distribution of HLA class I and II alleles (37, 38). Therefore, to maximize the potential use of our construct across populations, the HLA “supertype” alleles were considered as they are representative of the most frequent HLAs class I and II alleles worldwide. For HLA class -I, these are: HLA-A*01:01 (A1), HLA-A*26:01 (A1), HLA-A*02:01 (A2), HLA-A*03:01 (A3), HLA-A*24:02 (A24), HLA-B*07:02 (B7), HLA-B*08:01 (B8), HLA-B*27:05 (B27), HLA-B*39:01 (B27), HLA-B*40:01 (B44), HLA-B*58:01 (B58) and HLA-B*15:01 (B62) (39). And for HLA class-II: DRB1*03:01, DRB1*04:01, DRB1*04:05, DRB1*08:02, DRB1*11:01, DRB1*13:02 and DRB3*01:01 and DRB3*02:02 (9, 10, 40).

### Prediction and selection of candidate epitopes

#### Prediction of CTL- and HTL-epitopes

Epitopes of 8, 9, and 10 amino acids (aa) were predicted in *C. perfringens* proteins for the HLA class-I supertype alleles using NetMHCpan v4.1 (27) and MHCFlurry 2.0 (28), selecting based on the consensus of these predictors. For HLA class-II, epitopes of 15 aa were predicted using the NetMHCIIpan v4.0 (26). A threshold of rank ≤ 2% was used for all these software. With the prediction results, the epitope promiscuity (number of HLA alleles each epitope is predicted to bind to) was calculated, identifying epitopes that can bind to many alleles.

#### Discarding epitopes present in the host proteome

A previous study has shown that epitopes present in both proteins of the pathogen and proteins of the host may trigger an auto-immune response (41). To prevent this, all predicted epitopes matching human proteins with 100% identity and sequence coverage were removed.

In the first and second approaches described in 2.1.1, the whole protein was discarded. In the third approach, just the epitope was discarded. All the proteoforms of the human proteome (UniProt ID: UP000005640) were used as reference.

### Multi-epitope vaccine design

#### Identification of nested epitopes

To keep the artificial multi-epitope protein small while maximizing the number and quality of epitopes in the construct, highly immunogenic overlapping candidate epitopes were selected, which we called “nested epitopes”. These were defined as linear HTL-epitopes containing linear CTL-epitopes in its sequence. Previous studies have also used this approach to induce both CD4+ and CD8+ T-cell responses (40, 41).

#### Filtering proteins by the number of transmembrane helices and UniProt evidence

To further filter the list of nested epitopes, only predicted epitopes belonging to proteins having two or more predicted external transmembrane regions with epitopes within were considered. Transmembrane helices were predicted using TMHMM (http://www.cbs.dtu.dk/services/TMHMM/) (42, 43), and the presence of epitopes in external regions was checked with a custom script in Python. Additionally, only proteins with the following evidence categories according UniProt were considered: “Experimental evidence at protein level”, “Experimental evidence at transcript level” and “Protein inferred from homology”. The annotation found in UniProt was independently verified for correctness.

#### Selection of nested epitopes with high immunogenicity

From the proteins filtered above, only those nested epitopes predicted to bind 5 or more HLA-II supertype alleles were selected. Adjacent nested epitopes with overlap were merged to reduce the number of peptides while extending the predicted immunogenic regions. Non-overlapping nested epitopes were discarded.

#### Determination of highly conserved epitopes

Pathogens frequently mutate as an adaptation mechanism to environmental and immunological pressure, generating multiple variants (44). Selecting conserved regions may extend the validity of vaccines over time and confer protection against different strains. For this purpose, we performed BLASTp of our nested epitopes against *C. perfringens*. Epitopes with 90% of conservation or higher in the alignment were selected as candidate epitopes.

In addition, the conservation of each candidate nested epitope among the five *C. perfringens* toxinotypes was calculated. For each of the proteins having any of our candidate nested epitopes, a sequence alignment of the protein and its corresponding homologous proteins in the other toxinotypes was made. First, to find the homologous proteins, a Blast alignment was performed between the *C. perfringens* type A protein and the whole proteome of each toxinotype (**Supplementary Table 2**). Next, multiple global alignments were performed by protein using ClustalX2 (45). The alignments were visualized using CLC Sequence Viewer, where the regions corresponding to nested epitopes were extracted.

#### Design of multi-epitope vaccine candidates

Multi-epitope constructs were designed by concatenating the candidate epitopes in different orders. However, when assembling the constructs, neoepitopes that can bind to the HLA supertype alleles may appear at the interface of two candidate epitopes joined. Therefore, we need to know which epitope connections are allowed. This problem was modeled using a directed graph, where the nested candidate epitopes were represented as nodes, connected by directed edges representing the order of precedence in which the epitopes can be concatenated. To determine which directed edges are allowed in the graph, all the candidate epitopes were concatenated in a “pyramidal order”, which is a single sequence containing all possible connections (see **Figure 4A**). This sequence was submitted to NetMHCIIpan to predict if there are strong binder epitopes located in the interface between two nested epitopes. Thus, the edges (connections) harboring unwanted neoepitopes were removed from the directed graph. Then, all the semi and complete Hamiltonian paths were found using EpiSorter, a python-based toolset for multi-epitope assembly (**Figure 4B**). Finally, those candidate epitopes either disconnected from the rest of the graph or with no edges enabling the generation of a complete path were discarded.

Additionally, neoepitopes at the interface of two candidate epitopes may be present in the host proteome, which may result in unwanted immune responses potentially leading to auto-immune reactions or tolerance. To prevent this, the multi-epitope constructs were sliced in all the possible fragments of 15 aa and we evaluated if they were present in any human protein using BLASTp against the human proteome. This was performed using BioPython and the following parameters: program = “blastp”, database = “nr”, entrez_query = “txid9606ORGN, expect = 20000, alignments = 100”. For any 15-mer, if scores of 100% of coverage and 100% of identity were obtained, the corresponding construct was discarded.

### Structural analysis of the vaccine constructs

#### Prediction of physicochemical properties

The physicochemical properties of the multi-epitope vaccines were calculated using ProtParam (https://web.expasy.org/protparam/) (46), which computes the molecular weight, theoretical isoelectric point, grand average of hydropathicity (GRAVY), among other metrics.

#### Structural modeling

The structure of the multi-epitope proteins were predicted with AlphaFold v. 2.1.0 (47), using all the databases to search for templates and the model “monomer_casp14” to infer the 3D coordinates. For each multi-epitope construct, its possible 3D structures were ranked by mean pLDDT and the best five structures were refined by restrained energy minimization with AMBER99SB, as implemented in the AlphaFold2 pipeline. Thus, selecting the structure with highest confidence for each multi-epitope construct. Then, the two multi-epitope proteins with the highest mean pLDDT were selected to analyze their structural conformation through MD simulation.

#### Molecular dynamics

We performed 1.2 μs of MD for each multi-epitope structure. First, the structures were submitted to the PDB2PQR web server (48) to add the corresponding hydrogens at pH 7.4. Then, MD was carried out using NAMD 2.14 (49) with the CHARMM36 force field (50). Accordingly, NaCl ions were included on the surface of the protein based on its Coulombic potential using the package cIonize 2.0 (51). Next, ions located 20 Å farther from the protein were removed. Finally, the protein was solvated in a size-optimized box with 15 Å of padding and a salt concentration of 0.154 M, using the Autoionize Plugin v.1.5 (https://www.ks.uiuc.edu/Research/vmd/plugins/autoionize/).

Initially, only the water atoms were minimized for 5000 steps using the conjugate gradient algorithm. Then, MD of these atoms was performed at 0 K for 30 ps. Next, the whole system was minimized for 5000 steps, equilibrating the temperature to 298.15 K and the pressure 1 bar, as previously described (52). For this process, the Langevin thermostat and the Nosé-Hoover Langevin barostat were used in the NPT ensemble. Briefly, the system was heated from 50 K to 298 K, increasing the temperature by 4 K every 10 ps and applying harmonic restraints to the backbone with a force constant of 5 kcal/mol. Subsequently, the restraints were reduced by 10% every 0.05 ns. Finally, 1.2 μs of unrestrained MD of the NPT ensemble was performed at 298.15 K. Changes of temperature, potential energy, and density along the simulation were examined to verify convergence. All the processes described were performed in periodic boundary conditions with an integration time of 2 fs/timestep and Particle Mesh Ewald (PME) grid spacing of 1.0 Å. The cut-off for non-bonded interactions was set at 12 Å.

#### Assessing conformational convergence

For each trajectory, the alpha carbon root mean square deviation (Cα-RMSD) of all frames was calculated with an in-house Tcl script that uses VMD functions and the module “bigdcd”. The frame 0 was used as reference point. The structural compactness was quantified by the radius of gyration (Rg), which was calculated using an in-house Tcl script similar to the one described above. Trajectories where the RMSD and Rg did not converge were discarded. The remaining trajectories were trimmed to only include the interval where the RMSD and Rg converged.

It is known that two completely different structures could have the same Cα-RMSD when compared against the same reference. Therefore, we complemented our graphical approach with a Cα-RMSD-based clustering in Wordom v.0.22 (53). This method assigns conformations to the same cluster if every pair in the group has a Cα-RMSD less or equal to a given threshold (cluster diameter) (54), which we set as 2.5 Å. Therefore, finding a cluster much bigger than the rest suggests that a dominant conformation was produced by the simulation, suggesting conformational convergence.

#### Final refinement and evaluation of the structural quality

The centroid of the most populated cluster of each remaining multi-epitope MD was subjected to a two-step final refinement. First, main-chain and fast all-atom energy minimizations were conducted using the web server ModRefiner (55), without a reference structure. Second, the protein was solvated, followed by a minimization and MD of only water atoms using the methodology described above. Then, the output was minimized for 5000 steps in explicit solvent with NAMD and the CHARMM36 force field, applying harmonic restraints to all protein atoms with a force constant of 1 kcal/mol. The refined structures were uploaded to the web server MolProbity to construct Ramachandran plots. In addition, the web implementations of ProSA (56) and ERRAT (57) were used to further assess the structural quality.

#### Analysis of epitope flexibility and accessibility in MEP_12 structure

For each MD of the multi-epitope constructs, the centroids of the five most populated clusters were compared against each other. First, to have all the structures in the same coordinate system, all the centroids were RMS-aligned against the initial AlphaFold2 model in Pymol v.2.4.1 (58). Then, the AlphaFold2 model was removed and Pymol selection algebra was used to obtain a list of residues forming the same secondary structure in all the centroids.

To identify the most rigid and accessible epitope within the multi-epitope construct, the convergent trimmed trajectories were analyzed, identifying the most flexible protein regions by computing the Cα-RMSF of the trimmed trajectory in VMD. The trimmed trajectory was loaded with a step of 5 and aligned against frame 0, then the Cα-RMSF measured using these modified coordinates. Finally, the Solvent Accessible Surface Area (SASA) per epitope was measured during the trimmed trajectory using an in-house Tcl script. Then, the module “bigdcd” was used to load the complete trimmed trajectory and examine the changes of SASA along it, assessing the convergence of each epitope. For each epitope whose SASA converged, the mean SASA over the entire trajectory was computed and 95% confidence intervals calculated by computing the statistical inefficiency with the block averaging approach (59).

#### Evaluation of MEP_12 innate response potential

The potential of MEP_12 to trigger an innate response was tested based on its interaction with TLR1/TLR2 and TLR4/MD2. The representative conformation of MEP_12, from MD Ca-RMSD-based clustering, was docked against the ectodomains of TLR1/TLR2 (PDB ID: 2Z7X) and TLR4/MD-2 (TLR4/MD-2, PDB ID: 3FXI) using Haddock v.2.4 (60). The structures of these TLRs were submitted to PDBfixer to complete the missing sections and remove irrelevant heteroatoms (61). As TLR ectodomains are glycosylated *in vivo* (62), the covalently attached glycans of the structures were kept in order to obtain a TLR-MEP_12 interaction closer to real conditions.

As information about the possible interaction sites was not available, a blind docking approach was followed. Haddock ab-initio mode was used to scan the surface of MEP_12 and the corresponding TLR ectodomain, to find the most favorable interacting pose. This was performed during the rigid docking phase. Fifty thousand structures were computed for this step, to ensure that the whole surface of each protein will be sampled. Then, the best 500 structures were selected based on the Haddock scores, which were calculated from the semi-flexible simulated annealing and the final energy minimization refinement. The solutions of both docking steps were clustered by Fraction of Common Contacts (FCC), using 0.6 Å as threshold. Cluster-mean Haddock scores were computed using the structures with the four lowest values. Then, the clusters were ranked, where those with lower mean Haddock scores were the most favorable.

The most favorable clusters that had overlapping error bars (one standard deviation) of their Haddock-scores were selected. Protein-protein interaction analysis of the epitopes was performed using a custom Python pipeline, evaluating the plausibility of the solution based on prior structural data. After selecting one cluster, its most favorable structure was submitted to the web servers PDBsum and PRODIGY to map the interacting residues and to compute the protein-protein binding energy, respectively (63, 64).

#### Simulating the immune response

The immune response profile by immunization with the multi-epitope vaccine was simulated using C-IMMSIM (https://kraken.iac.rm.cnr.it/C-IMMSIM/). Three vaccinations with a dose of 1000 unit of the multi-epitope vaccine and 100 of adjuvant without LPS were administrated as described in **Figure 10A**. The most frequent HLA alleles were considered: HLA-A*0101, HLA-B*08:01, HLA-B*15:01, DRB1-11:01 and DRB3-02:02. One thousand timesteps of 8 hours were simulated, representing ~11 months. Vaccine doses were administered four weeks apart, as commonly recommended (65), corresponding to days 3, 30 and 60. Pathogen challenge was introduced on day 111 by inoculating 1000 units of those proteins of *C. perfringens type A* harboring epitopes of the vaccine, with a pathogen multiplication factor of 0.2 (**Figure 10A**).

#### Multi-epitope sequence design and codon optimization

Codon optimization was performed to improve the expression efficiency of the vaccine construct in *Escherichia coli* for production. The codon usage table of *E. coli* K12 strain, available in the Codon Usage Database (https://www.kazusa.or.jp/codon/), was used for reverse translation. The CAIcal SERVER (http://genomes.urv.es/CAIcal/) was used to calculate the Codon Adaptation Index (CAI). The cDNA sequence obtained was analyzed with NEBcutter (http://nc2.neb.com/NEBcutter2/), identifying cleavage sites of commercially available restriction enzymes.

## Results

### Identification of proteins with high epitope density

**Figure 2:**
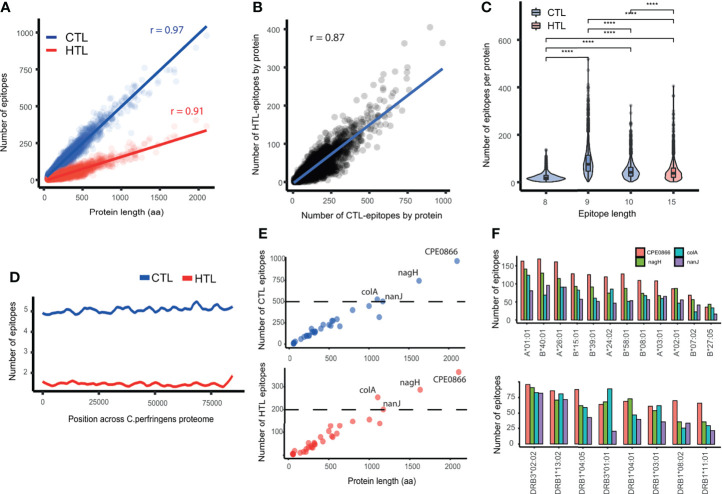
Description of the predicted epitopes in *C. perfringens*. **(A)** Correlation between the number of epitopes and protein length. Blue: CTL-epitopes. Red: HTL-epitopes. **(B)** Correlation between the number of HTL- and CTL-epitopes. **(C)** Comparison of the number of epitopes per protein, for different epitope lengths of CTL- (8, 9 and 10 aa) and HTL- (15 aa) epitopes. **(D)** Lasso regression of the number of CTL- (blue) and HTL- (red) epitopes across the proteome of *C. perfringens*. **(E)** Correlation between protein length and number of CTL- and HTL-epitopes in the protein (top and bottom, respectively). Proteins with more than 500 CTL- and 200 HTL-epitopes (above the dashed line) are labeled. **(F)** Bar plot showing the number of CTL- and HTL-epitopes (top and bottom, respectively) by HLA allele within, for the four proteins highlighted on panel **(E)**. **** means p-value < 0.0001.

A total of 429809 CTL- and 121450 HTL-epitopes were predicted from the proteome of *C. perfringens* Type A. The number of CTL- and HTL-epitopes predicted were strongly correlated with the protein length (CTL: p < 2.2e-16, r = 0.97; HTL: p < 2.2e-16, r = 0.91, Pearson correlation) (**Figure 2A**). There is also a positive correlation between the number of CTL- and HTL-epitopes predicted by protein (p < 2.2e-16, r = 0.87, Pearson correlation) (**Figure 2B**), indicating that *C. perfringens* proteins containing a higher number of predicted CTL-epitopes tend to contain more HTL-predicted epitopes as well.

Regarding the epitope length, we found a significantly greater number of 9-mer than 8-mer or 10-mer predicted CTL-epitopes (Wilcoxon test, p value < 2.2e-16), as biologically expected. Additionally, the number of 9-mer CTL epitopes predicted by protein was higher than the number of HTL-epitopes (**Figure 2C**). These observations were statistically significant even when normalizing by protein length (Wilcoxon test, p < 2.2e-16) (**Supplementary Figure 1A**).

The distribution of predicted epitopes across the proteome was explored by randomly concatenating the sequences of all the 2721 proteins and calculating the epitope density along it. Local peaks were detected, showing that the predicted epitopes were not evenly distributed across the proteome and that not all proteins have the same epitope density (**Figure 2D**).

Epitopes that were either duplicated (i.e. appearing in two or more different proteins) or present in any human protein were removed, resulting in 121244 (99.83%) HTL- and 427944 (99.56%) predicted CTL-epitopes left (**Supplementary Table 3**). This comprises 59578, 237664 and 130702 CTL-epitopes predicted of 8, 9 and 10 aa, respectively (**Supplementary Figure 1B**). Regarding the epitope promiscuity (i.e. the number of alleles an epitope can bind to), we found that the majority of the CTL-epitopes predicted (312032 out of 427944) bind to only one HLA-I allele. Similarly, 67080 of the 121244 HTL-epitopes were predicted to bind just one HLA-II. Noteworthy, there were 2 promiscuous CTL-epitopes binding to 11 HLA-I alleles, and 33 HTL-epitopes binding to 8 HLA-II alleles (**Supplementary Figures 1C, D**).

The evidence status of the proteins with at least 1 epitope predicted was retrieved from UniProt, obtaining 32 with “evidence at protein level”, 1 with “evidence at transcript level”, 1064 with “inferred from homology”, 1623 “predicted”, and 1 “uncertain”. The set of proteins with “evidence at protein level”, when compared with the set of proteins “inferred from homology”, showed no significant difference in the number of epitopes (p = 0.33) but in epitope density (p < 0.02) (**Supplementary Figures 1E, F**).

Among the proteins with “evidence at protein level”, 25 show an epitope density above 0.5 (**Supplementary Table 4**). Notoriously, each of the proteins Collagenase A, Exo-alpha-sialidase, alpha-n-acetylglucosaminidase and hyaluronoglucosaminidase contain more than 500 CTL- and 200 HTL-epitopes (**Figure 2E**), and more than 15 epitopes per HLA supertype allele (**Figure 2F**).

### Evaluation of the HTL-epitopes in *C. perfringens* toxins

**Figure 3:**
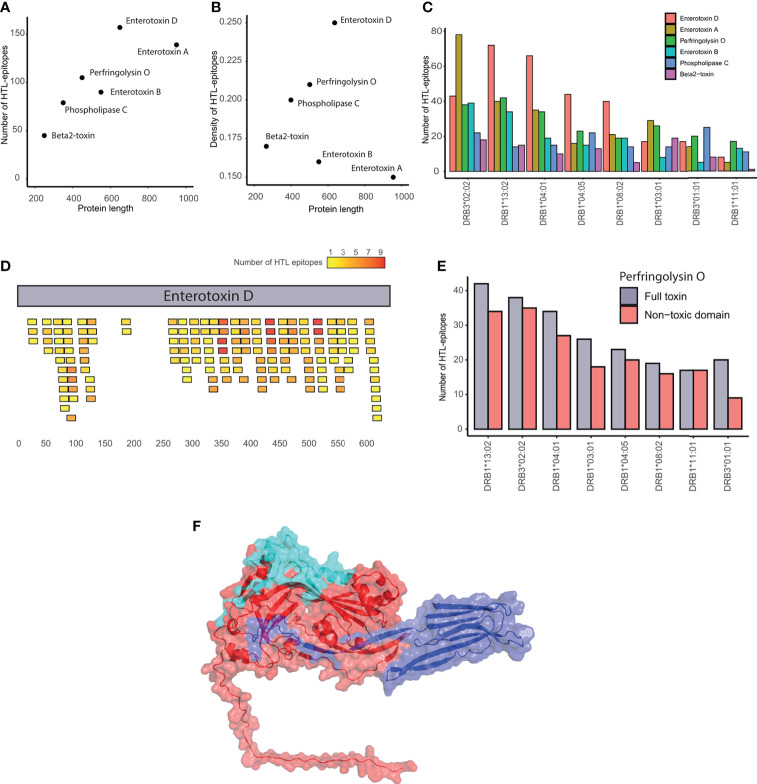
Evaluation of epitopes in *C. perfringens* toxins. **(A)** Correlation between the number of HTL-epitopes and protein length. **(B)** Correlation between HTL-epitope density and protein length. **(C)** Number of HTL-epitopes within toxins binding each of the HLA-II supertype alleles. **(D)** Location of the HTL-epitopes along the enterotoxin **(D)** Epitopes are colored in a gradient from yellow to red, representing the number of HLA alleles they bind. **(E)** Number of HTL-epitopes in the whole Perfringolysin O toxin (in gray) and its non-toxic domain (red) that are predicted to bind each of the HLA-II supertype alleles. **(F)** Structure of the Perfingolysin O. The toxic-domain is represented in blue. The non-toxic domain in red, highlighting the most promiscuous epitopes in cyan.

The six toxins evaluated have a different number (45–157) but similar density (0.15-0.25) of HTL-epitopes. Notably, enterotoxin D contains the highest number of HTL-epitopes even not being the largest toxin. Contrarily, beta2-toxin showed the lowest number of HTL-epitopes (**Figure 3A**). In terms of HTL-epitope density, the enterotoxins A and D showed the lowest and highest values, respectively (**Figure 3B**).

All the toxins, except the beta2-toxin, contained several epitopes by HLA allele. Among the HLA-II alleles used, DRB3*02:02 and DRB1*11:01 were found to recognize the highest and the lowest number of epitopes from toxins (**Figure 3C**). Regions enriched with promiscuous HTL-epitopes within the toxins were identified. For example, the C-terminal region of the enterotoxin D contains the most promiscuous epitopes of the protein, while the region between positions 130 and 256aa has almost no epitopes (**Figure 3D**). Similarly, the HTL-epitopes of the enterotoxins A and B were mainly located at the N-terminal region. Additionally, the non-toxic domain of Perfringolysin O contains at least nine predicted epitopes per HLA-II allele (**Figure 3E**). Furthermore, among the toxins, we identified 12 HTL-epitopes binding five or more HLA-II alleles, covering the majority of HLA-II supertype alleles (**Table 1**). We also found that four of these promiscuous epitopes are located in the external region of the non-toxic domain of the Perfringolysin O (**Figure 3F**). Altogether, it suggests that this domain can be considered as a potential subunit-based vaccine.

**Table 1:** Promiscuous HTL-epitopes in *C. perfringens* toxins.

Epitopes	Alleles	Number of Alleles	Proteins
PENIKIIANGKVVVD	DRB1*03:01, DRB1*04:01, DRB1*04:05, DRB1*08:02, DRB1*11:01, DRB1*13:02, DRB3*02:02	7	PHOSPHOLIPASE C
PKYIVIHDTDNRQAG	DRB1*03:01, DRB1*04:01, DRB1*04:05, DRB1*08:02, DRB1*13:02, DRB3*01:01	6	ENTEROTOXIN D
RKPININIDLPGLKG-NPKYIVIHDTDNRQA	DRB1*03:01, DRB1*04:01, DRB1*04:05, DRB1*08:02, DRB1*13:02, DRB3*02:02	6	PERFRINGOLYSIN O - ENTEROTOXIN D
MLEEFKYDPNQQLKS-LEEFKYDPNQQLKSF	DRB1*03:01, DRB1*04:01, DRB1*04:05, DRB1*13:02, DRB3*01:01, DRB3*02:02	6	BETA2 TOXIN
LKSFEILNSQKIDNK	DRB1*04:01, DRB1*04:05, DRB1*08:02, DRB1*11:01, DRB1*13:02, DRB3*02:02	6	BETA2 TOXIN
KYIVIHDTDNRQAGA	DRB1*03:01-DRB1*04:01-DRB1*04:05, DRB1*13:02-DRB3*01:01	5	ENTEROTOXIN D
KRKPININIDLPGLK	DRB1*03:01, DRB1*04:01, DRB1*04:05, DRB1*13:02, DRB3*02:02	5	PERFRINGOLYSIN O
EIRKVIKDNATFSTK-IRKVIKDNATFSTKN-NDNINIDLSNSNVAV-EMLEEFKYDPNQQLK-EEFKYDPNQQLKSFE	DRB1*03:01, DRB1*04:01, DRB1*13:02, DRB3*01:01, DRB3*02:02	5	PERFRINGOLYSIN O - PERFRINGOLYSIN O - ENTEROTOXIN A - BETA2 TOXIN - BETA2 TOXIN
GEIFNIDGKEGSWYK	DRB1*03:01, DRB1*08:02, DRB1*11:01, DRB1*13:02, DRB3*01:01	5	ENTEROTOXIN B
ENIKIIANGKVVVDK-NIKIIANGKVVVDKD	DRB1*0301, DRB1*08:02, DRB1*11:01, DRB1*13:02, DRB3*02:02	5	PHOSPHOLIPASE C
WNEKYSSTHTLPART-NEKYSSTHTLPARTQ-GSNYGVIGTLRNNDK-ASKSYITIVNEGSNN-SKSYITIVNEGSNNG	DRB1*04:01, DRB1*04:05, DRB1*08:02, DRB1*11:01, DRB3*02:02	5	PERFRINGOLYSIN O - PERFRINGOLYSIN O - ENTEROTOXIN D - ENTEROTOXIN D - ENTEROTOXIN D
KQGIVKVNSALNMRS-KSFEILNSQKIDNKE	DRB1*04:01, DRB1*04:05, DRB1*08:02, DRB1*13:02, DRB3*02:02	5	ENTEROTOXIN D - BETA2 TOXIN

### Design of multi-epitope vaccine candidates

We identified 112714 nested epitopes from the whole proteome, comprising 112714 predicted HTL-epitopes containing 145854 predicted CTL-epitopes (**Supplementary Table 5**). Next, from the 2685 proteins containing nested epitopes, 266 proteins had at least two external transmembrane regions with at least one nested epitope. Then, the proteins annotated as “predicted proteins” or “uncertain proteins” were removed, resulting in 105 proteins containing 1884 nested epitopes. From this set, only nested epitopes that can bind to at least five HLA-II alleles were selected, obtaining 42. And it was possible to merge 29 of these 42 nested epitopes into 11 overlapped nested epitopes (**Table 2**), which represent the candidates for the design of the muti-epitope construct (**Figure 1C**). The conservation analysis showed that these candidate epitopes were highly conserved among the sequences of the different strains of C. perfringens (**Table 2**). Moreover, these epitopes are 100% conserved among the five toxinotypes (**Supplementary Figure 2**).

**Table 2:** Characteristics of the predicted epitopes selected for the construction of the multi-epitope protein.

Epitope	Overlaped epitope	Nested_epitope	HLA-II alleles	Protein name	Protein ID	Position	Epitope	HLA-I Alleles	Conservation
Ep_0	IDGKEYKIANNALIGEGK	IDGKEYKIANNALIG	DRB1*13:02, DRB3*02:02, DRB1*04:05, DRB1*08:02, DRB1*04:01	FtsX domain-containing protein	Q8XM39	453	EYKIANNALI	A*24:02	160/161
							KEYKIANNAL	B*40:01	
							EYKIANNAL	A*24:02, B*08:01, B*39:01	
							YKIANNALI	B*39:01	
							KEYKIANNA	B*40:01	
		DGKEYKIANNALIGE	DRB1*13:02, DRB1*11:01, DRB3*02:02, DRB1*04:05, DRB1*08:02, DRB3*01:01, DRB1*04:01			454	EYKIANNALI	A*24:02	
							KEYKIANNAL	B*40:01	
							EYKIANNAL	A*24:02, B*08:01, B*39:01	
							YKIANNALI	B*39:01	
							KEYKIANNA	B*40:01	
		GKEYKIANNALIGEG	DRB1*13:02, DRB1*11:01, DRB3*02:02, DRB1*04:05, DRB1*08:02, DRB3*01:01, DRB1*04:01			455	EYKIANNALI	A*24:02	
							KEYKIANNAL	B*40:01	
							EYKIANNAL	A*24:02, B*08:01, B*39:01	
							YKIANNALI	B*39:01	
							KEYKIANNA	B*40:01	
		KEYKIANNALIGEGK	DRB1*13:02, DRB3*02:02, DRB1*04:05, DRB1*08:02, DRB1*04:01			456	EYKIANNALI	A*24:02	
							KEYKIANNAL	B*40:01	
							EYKIANNAL	A*24:02, B*08:01, B*39:01	
							YKIANNALI	B*39:01	
							KEYKIANNA	B*40:01	
Ep_1	LYEKGFLHAKTIVADSS	LYEKGFLHAKTIVAD	DRB1*11:01, DRB3*02:02, DRB1*04:05, DRB1*08:02, DRB1*04:01	Cardiolipin synthase	P0C2E2	387	FLHAKTIV	B*08:01	89/89
							LHAKTIVA	B*39:01	
							FLHAKTIVA	A*02:01, B*08:01	
		YEKGFLHAKTIVADS	DRB1*11:01, DRB3*02:02, DRB1*04:05, DRB1*08:02, DRB1*04:01			388	FLHAKTIV	B*08:01	
							LHAKTIVA	B*39:01	
							FLHAKTIVA	A*02:01, B*08:01	
		EKGFLHAKTIVADSS	DRB1*13:02, DRB1*11:01, DRB3*02:02, DRB1*04:05, DRB1*08:02, DRB1*04:01			389	FLHAKTIV	B*08:01	
							LHAKTIVA	B*39:01	
							FLHAKTIVA	A*02:01, B*08:01	
Ep_2*	EGKIVVIIDNSPSVIIL	EGKIVVIIDNSPSVI	DRB1*13:02, DRB3*02:02, DRB1*04:05, DRB1*03:01, DRB3*01:01, DRB1*04:01	Stage V sporulation protein AF	Q8XLQ7	245	VIIDNSPSV	A*02:01, A*26:01	85/86
							IIDNSPSVI	A*02:01	
		GKIVVIIDNSPSVII	DRB1*13:02, DRB3*02:02, DRB1*04:05, DRB1*03:01, DRB3*01:01, DRB1*04:01			246	VIIDNSPSV	A*02:01, A*26:01	
							IIDNSPSVI	A*02:01	
		KIVVIIDNSPSVIIL	DRB1*13:02, DRB3*02:02, DRB1*03:01, DRB3*01:01, DRB1*04:01			247	VIIDNSPSV	A*02:01, A*26:01	
							IIDNSPSVI	A*02:01	
							DNSPSVIIL	B*39:01	
Ep_3	GAERFVLISTDKAVNPT	GAERFVLISTDKAVN	DRB1*11:01, DRB3*02:02, DRB1*04:05, DRB1*08:02, DRB1*04:01	Polysacc synt 2 domain-containing protein	Q8XN75	406	FVLISTDKAV	A*02:01	30/32
							ERFVLISTDK	B*27:05	
							VLISTDKAV	A*02:01	
							AERFVLIST	B*40:01	
		AERFVLISTDKAVNP	DRB1*13:02, DRB1*11:01, DRB3*02:02, DRB1*04:05, DRB1*08:02, DRB1*04:01			407	FVLISTDKAV	A*02:01	
							ERFVLISTDK	B*27:05	
							VLISTDKAV	A*02:01	
							AERFVLIST	B*40:01	
		ERFVLISTDKAVNPT	DRB1*13:02, DRB1*11:01, DRB1*04:05, DRB1*08:02, DRB1*04:01			408	FVLISTDKAV	A*02:01	
							ERFVLISTDK	B*27:05	
							VLISTDKAV	A*02:01	
Ep_4	IKENEFVVDGSTRLSDL	IKENEFVVDGSTRLS	DRB1*04:01, DRB3*01:01, DRB3*02:02, DRB1*03:01, DRB1*13:02	Probable hemolysin-related protein	Q8XPD3	339	FVVDGSTRL	A*02:01, A*26:01	41/41
		KENEFVVDGSTRLSD	DRB1*04:01, DRB3*01:01, DRB3*02:02, DRB1*03:01, DRB1*13:02			340	FVVDGSTRL	A*02:01, A*26:01	
		ENEFVVDGSTRLSDL	DRB1*13:02, DRB3*02:02, DRB1*03:01, DRB3*01:01, DRB1*04:01			341	FVVDGSTRL	A*02:01, A*26:01	
							DGSTRLSDL	B*08:01	
Ep_5	RHKDKIYIDTSPVNNLI	RHKDKIYIDTSPVNN	DRB1*04:01, DRB1*04:05, DRB3*01:01, DRB3*02:02, DRB1*03:01, DRB1*13:02	TraG-D C domain-containing protein	Q93M96	158	KIYIDTSPV	A*02:01	70/82
		HKDKIYIDTSPVNNL	DRB1*13:02, DRB3*02:02, DRB1*04:05, DRB1*03:01, DRB3*01:01, DRB1*04:01			159	YIDTSPVNNL	A*02:01	
							KIYIDTSPV	A*02:01	
							IDTSPVNNL	B*40:01	
		KDKIYIDTSPVNNLI	DRB1*13:02, DRB3*02:02, DRB1*03:01, DRB3*01:01, DRB1*04:01			160	YIDTSPVNNL	A*02:01	
							KIYIDTSPV	A*02:01	
							DTSPVNNLI	A*26:01	
							IDTSPVNNL	B*40:01	
Ep_6	ASATYYIDEDSKIKTA	ASATYYIDEDSKIKT	DRB3*02:02, DRB1*04:05, DRB1*03:01, DRB3*01:01, DRB1*04:01	FtsX domain-containing protein	Q8XM39	331	ATYYIDEDSK	A*03:01	126/128
							TYYIDEDSKI	A*24:02	
							YIDEDSKIK	A*01:01	
							YYIDEDSKI	A*24:02	
		SATYYIDEDSKIKTA	DRB3*02:02, DRB1*04:05, DRB1*03:01, DRB3*01:01, DRB1*04:01			332	ATYYIDEDSK	A*03:01	
							TYYIDEDSKI	A*24:02	
							YIDEDSKIK	A*01:01	
							YYIDEDSKI	A*24:02	
Ep_7*	VPDNIVSNLKPIANKI	VPDNIVSNLKPIANK	DRB1*13:02, DRB1*11:01, DRB3*02:02, DRB1*03:01, DRB1*08:02	FtsX domain-containing protein	Q8XM39	490	VSNLKPIANK	A*03:01	136/147
							VPDNIVSNL	B*07:02, B*08:01, B*39:01	
							SNLKPIANK	A*03:01	
		PDNIVSNLKPIANKI	DRB1*13:02, DRB1*11:01, DRB3*02:02, DRB1*03:01, DRB1*08:02			491	VSNLKPIANK	A*03:01	
							SNLKPIANK	A*03:01	
							NLKPIANKI	B*08:01	
Ep_8	LDYKFILDTNYIEAKL	LDYKFILDTNYIEAK	DRB3*02:02, DRB1*04:05, DRB1*03:01, DRB3*01:01, DRB1*04:01	Spore germination protein KA	Q8XMP0	191	FILDTNYIEA	A*02:01	42/43
							ILDTNYIEAK	A*03:01	
							ILDTNYIEA	A*03:01	
							KFILDTNYI	A*24:02	
							YKFILDTNY	B*15:01	
		DYKFILDTNYIEAKL	DRB3*02:02, DRB1*04:05, DRB1*03:01, DRB3*01:01, DRB1*04:01			192	FILDTNYIEA	A*02:01	
							ILDTNYIEAK	A*03:01	
							ILDTNYIEA	A*03:01	
							KFILDTNYI	A*24:02	
							DTNYIEAKL	A*26:01	
							YKFILDTNY	B*15:01	
Ep_9	LDDFITIEKANNSYTF	LDDFITIEKANNSYT	DRB1*13:02, DRB1*11:01, DRB3*02:02, DRB1*08:02, DRB1*04:01	Cardiolipin synthase	Q8XP94	265	ITIEKANNSY	A*01:01, A*26:01, B*15:01, B*58:01	114/115
							TIEKANNSY	A*01:01, A*26:01, B*15:01	
		DDFITIEKANNSYTF	DRB1*13:02, DRB1*11:01, DRB3*02:02, DRB1*08:02, DRB1*04:01			266	ITIEKANNSY	A*01:01, A*26:01, B*15:01, B*58:01	
							IEKANNSYTF	B*40:01	
							KANNSYTF	B*58:01	
							TIEKANNSY	A*01:01, A*26:01, B*15:01	
Ep_10	SDNDYVIVNTEGGEFD	SDNDYVIVNTEGGEF	DRB1*11:01, DRB3*02:02, DRB1*04:05, DRB1*08:02, DRB1*04:01	UPF0182 protein CPE0011	Q8XPF2	461	VIVNTEGGEF	B*15:01	173/174
							IVNTEGGEF	A*26:01, B*15:01	
		DNDYVIVNTEGGEFD	DRB1*13:02, DRB1*11:01, DRB3*02:02, DRB1*04:05, DRB1*08:02, DRB1*04:01			462	VIVNTEGGEF	B*15:01	
							IVNTEGGEF	A*26:01, B*15:01	

Epitopes “2” and “7” (with asterisk) were not included in the final design. Conservation is represented as the number of sequences where epitope is conserved over the total number of sequences analyzed.

**Figure 4:**
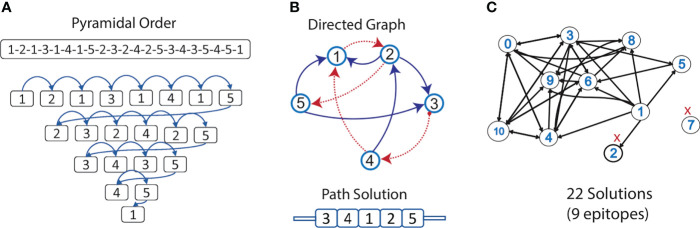
Selection of epitopes for the design of a multi-epitope construct. **(A)** An example of the pyramidal order for 5 epitopes, showing how they should be concatenated into a new protein, to evaluate the presence of neoepitopes in all the connections in just a single prediction step. **(B)** Allowed connections (without neoepitopes) are represented in the directed graph. The Hamiltonian path (in red) exemplifies a solution containing all the nodes. **(C)** Graph representing the nested epitopes (nodes) and its allowed connections (edges), selected in this study for the construction of the multi-epitope construct. Epitopes discarded from the design are marked in red.

These 11 overlapped nested epitopes were used to build the directed graph. By finding the allowed directed edges, epitopes “7” was discarded as it was disconnected from the rest of the graph. Also, epitope “2”, because there were no edges permitting the generation of a complete path (**Figure 4C**). From the subgraph containing the nine remaining overlapped nested epitopes, we obtained 21 different Hamiltonian paths, representing multi-epitope constructs (**Supplementary Table 6**). Using BLASTp, we obtained that only a 5 aa fragment of an epitope (DTNYI) matched with a human protein (ATP-dependent DNA helicase HFM1) with a 100% coverage and identity. Therefore, no construct was discarded.

### Structural analysis of the multi-epitope constructs

#### Physicochemical characterization

We evaluated the physicochemical properties of the constructs using ProtParam (**Supplementary Table 7**). Each one had 150 aa, a molecular weight of 16.83 kDa and a predicted pI of 4.61, indicating an acidic nature. The number of negatively and positively charged residues computed at pH 7 were 27 and 17, respectively. The aliphatic index (relative volume occupied by the aliphatic side chains) was 93, indicating a thermostable nature. The construct had a GRAVY value of -0.363.

#### Modelling the constructs

Models of each multi-epitope construct were prepared in AlphaFold2, and its mean pLDDT were computed. Values under 50 were obtained for all models (**Supplementary Figure 3A**), suggesting structural disorder (66). After modeling the 21 multi-epitope constructs, only MEP_6 and MEP_12 were selected, as they showed the highest mean pLDDT.

**Figure 5:**
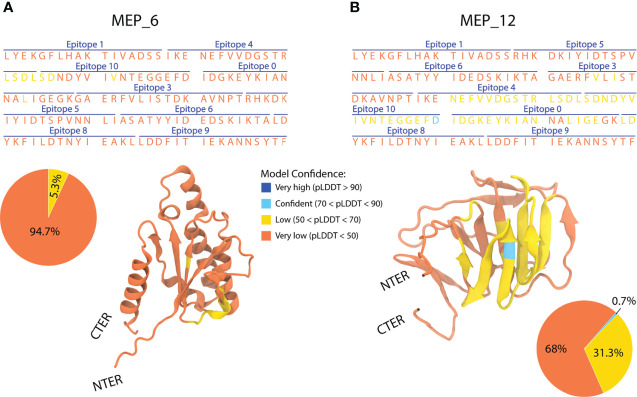
Amino acid sequence, epitope sorting and AlphaFold2 3D model of the multi-epitope constructs MEP_6 **(A)** and MEP_12 **(B)**. The confidence value (pLDDT) is categorized in 4 groups (orange, yellow, cyan, and blue), representing their percentage in pie charts.

The structures of the multi-epitope (MEP) MEP_6 and MEP_12 had the highest mean pLDDT values, being the most reliable ones. Besides the similarity in their mean pLDDT, the distribution of per-residue pLDDT notably differed. MEP_6 had 94.7% of its residues with pLDDT under 50 and 5.3% between 50 and 70 (**Figure 5A**). In contrast, MEP_12 had 68% of residues with pLDDT under 50, 31.3% between 50 and 70, and 0.7% between 70 and 90 (**Figure 5B**).

In addition, the per-residue pLDDT seemed to be non-dependent on the epitope from which the residues belong. For instance, the residues of epitope “4” had pLDDTs under 50 in MEP_6, whereas values between 50 and 70 in MEP_12 (**Figure 5**).

#### Assessing the conformational convergence

The confidence of our best candidate models (MEP_6 and MEP_12) were improved by performing extensive MDs to refine them, and their conformational changes were evaluated. The RMSD and Rg over each trajectory were measured to assess their conformational convergence.

**Figure 6:**
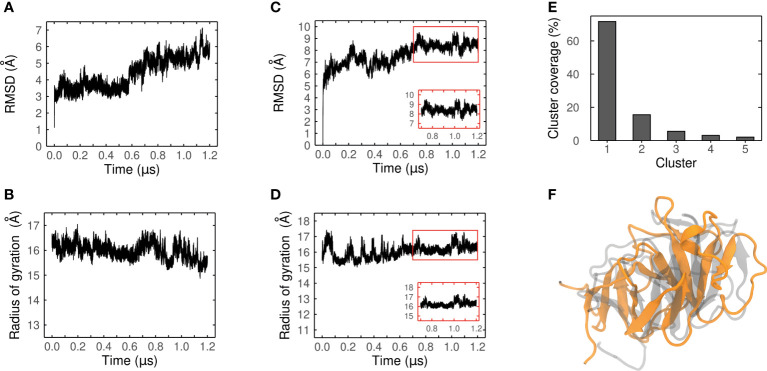
Convergence analysis of the MD simulation. Ca-RMSD values and radius of gyration (Rg) of MEP_6 **(A, B)** and MEP_12 **(C, D)** during a simulation of 1.2 μs. Inset plots in **(C, D)** show the last 500 ns where the RMSD and Rg converged. **(E)** Coverage (%) of the five most populated clusters obtained from the RMDS-based clustering. **(F)** Structural alignment between the AlphaFold2 model (gray) and the centroid of the most populated cluster (in orange) of MEP_12.

The RMSD of MEP_6 increased during the last 600 ns of MD, which is indicative of a conformation that is still changing (**Figure 6A**). The conformational instability was corroborated by an unstable Rg, which continuously decreased during all the trajectory (**Figure 6B**). Therefore, MEP_6 was discarded of further analysis as it did not reach conformational convergence.

In contrast, the RMSD and Rg of MEP_12 reached a steady state during the last 500 ns (**Figures 6C, D**) suggesting conformational convergence of the trajectory. It was verified by an RMSD-based clustering of frames of the last 500 ns, using 2.5 Å as threshold. More than 70% of the conformations adopted during this lapse were highly similar among them and were grouped in Cluster 1, confirming the convergence (see **Figure 6E**; **Supplementary Table 8**).

As the centroid of Cluster 1 represents the preferred conformation of MEP_12, it was retrieved and considered as the most probable average structure of MEP_12 in solution. Remarkably, although displaced due to the MD, the β-strands formed by the same residues are present both in the centroid and in the initial AphaFold2 model (**Figure 6F**).

#### Evaluating the structural quality

**Figure 7:**
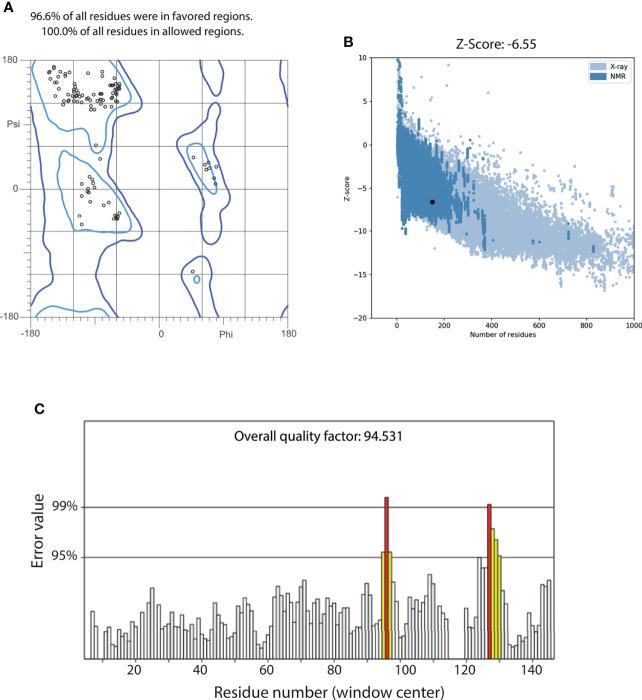
Quality assessment of the refined centroid of MEP_12 most populated cluster. **(A)** Ramachandran plot of MEP_12 centroid, indicating the percentage of residues in favored (light blue) and allowed (blue) regions. **(B)** Scatterplot of the Z-scores of MEP_12 centroid (black dot) and structures with experimental evidence obtained from NMR (blue) and X-ray crystallography (light blue). **(C)** ERRAT plot of MEP_12 centroid. Bars represent the error value (white: error < 95%, yellow: 95%, error < 99%, red: error > 99%) of a nine-residue sliding window. The overall quality factor indicates the percentage of protein residues with error values lower than 95%.

A two-steps refinement of the centroid was performed using ModRefiner and NAMD, obtaining a structure that remained close to the original conformation (**Supplementary Figure 3B**). The quality of the refined structure was assessed using the web servers MolProbity, ProSA and ERRAT. We observed that all the residues in the model were correctly oriented, as the Ramachandran plot indicated that 100% of residues were in allowed regions, not showing any outliers (**Figure 7A**). Furthermore, 96.6% of the residues were observed in favored regions. ProSA predicted a Z-score of -6.55 for our model, which is within the range of values observed in other proteins of similar length, obtained from NMR or X-ray crystallography (**Figure 7B**). An ERRAT overall score of 94.531 was obtained, indicating that this model has a good resolution. Notably, only windows centered at residues 96 and 127 had error values above 99% (**Figure 7C**). All the quality statistics were better in the refined structure than the original model (**Supplementary Figure 4**).

#### Analysis of MEP_12 trajectory

**Figure 8:**
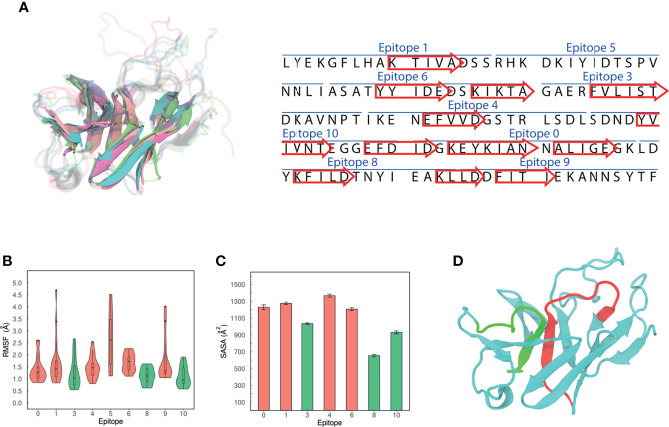
Structural characteristics of MEP_12. **(A)** Alignment of the 3D structures of MEP_12 from all the centroids of the five most populated clusters. Cyan, purple, orange, green, and pink cartoons correspond to the centroids of clusters 1, 2, 3, 4 and 5 respectively. The twelve β-strands in the structure (left) are represented as arrows in the sequence (right). **(B)** Violin plots showing the distribution of Ca-RMSF values. The lowest Ca-RMSF distributions are colored in green. **(C)** Mean SASA by epitope. The error bars represent 95% confidence intervals. **(D)** Modeled structure of MEP_12, showing epitopes “3” and “6” in red and green, respectively.

An ensemble approach was followed to assign the secondary structures of the aminoacidic residues. The centroids of the five most populated clusters were compared, as they are distinct and representative conformations of MEP_12 in solution. Then, sets of residues forming the same secondary structure in all the centroids were retrieved. We reasoned that if those sets are folded in the same way, even in different but representative conformations, there is a high chance that they might adopt that conformation when synthesized. Notably, comparing the five centroids, we found that 12 β-strands were formed by the same sets of residues (**Figure 8A**). The number of residues forming these β-strands represent 41.33% of the MEP_12. The other residues were mostly in loops in all the centroids evaluated. No consistent alpha helices were found.

By analyzing the Cα-RMSF, epitopes “3”, “8” and “10” were found to be the least flexible ones (**Figure 8B**). The rigidity of “3” can be explained because part of this epitope forms a parallel β-sheet with a portion of epitope “6” (**Figure 8D**). Next, the mean SASA over the steady state of the MD was computed, excluding epitope “4” as it was not stable over the MD (**Supplementary Figure 5**). Epitopes “3”, “10”, and “8” showed the lowest mean SASA values, in decreasing order (**Figure 8C**). Altogether, this indicates that epitope “3” is the most accessible among the rigid epitopes of MEP_12.

#### Evaluation of MEP_12 innate response potential

**Figure 9:**
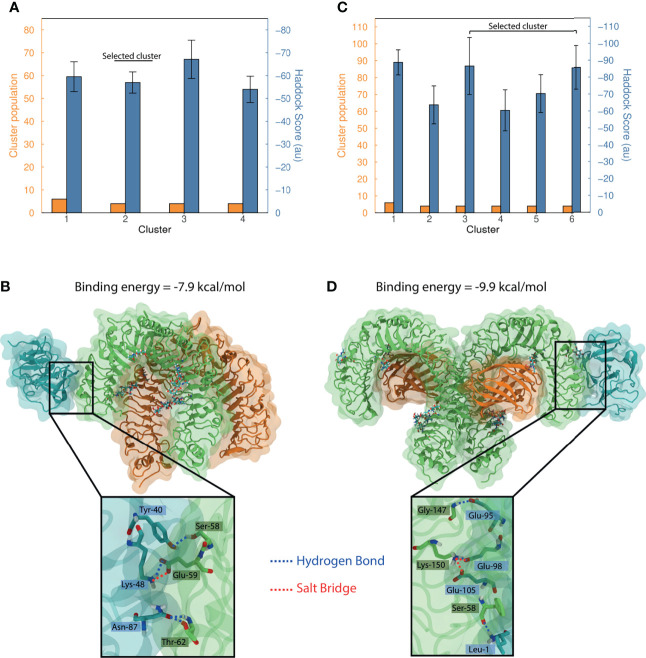
Protein docking of MEP_12 against TLR1/TLR2 and TLR4/MD-2. Bar plots summarizing the dockings **(A)** MEP_12-TLR1/TLR2 and **(C)** MEP_12-TLR4/MD2. The number of structures and the mean Haddock-score by cluster are shown in orange and blue, respectively. Whiskers in the mean Haddock-score bars represent one standard deviation. Structural representation of the most favorable binding mode of the cluster selected, of dockings **(B)** MEP_12-TLR1/TLR2, and **(D)** MEP_12-TLR4/MD2; showing the binding energy computed in PRODIGY. MEP_12 is colored in cyan; TLR1 and TLR4 in green; and TLR2 and MD2 in orange. Glycosylated residues and attached glycans (cyan) are shown as sticks, and non-carbon atoms are colored following the CPK convention. The inset plots show a closeup of the residues involved in polar interactions (as cyan and green sticks). The hydrogen bonds and salt bridges are represented by blue and red lines, respectively, connecting the interacting atoms which are labeled indicating amino acid and position.

Blind docking of MEP_12 against TLR1/TLR2 resulted in four clusters containing 3.6% of the refined structures. The Haddock-scores of these clusters showed overlapping error bars (one standard deviation) (**Figure 9A**), not allowing to discern which cluster is the best.

The TLR1/TLR2 crystal structure had missing residues in the N-termini of both TLRs. As the cluster “1” contained binding modes of MEP_12 interacting with TLR2 in a region that might be occupied by those missing residues, it was discarded (**Supplementary Figures 6A, B**). All the other clusters showed MEP_12 docked to similar regions of TLR1 (**Supplementary Figure 6A**). The best binding mode of the cluster “2” showed contacts with epitopes “3”, “4” and “6”. It showed four hydrogen bonds and one salt bridge that stabilized the interaction (**Figure 9B**). The best binding modes of the cluster “3” and “4” showed contacts were with epitopes “6” and “0”; and “0”, “1” and “9”, respectively (**Supplementary Table 9**).

Blind docking of MEP_12 against TLR4/MD-2 resulted in six clusters containing 5.2% of the refined structures. The Haddock-scores of the best four clusters had overlapping error bars (one standard deviation) (**Figure 9C**). From this set, only cluster “5” contained solutions in which MEP_12 interacted exclusively with the co-receptor MD-2, while the others showed MEP_12 interacting with any of the two chains of TLR4 (**Supplementary Figure 6C**). Therefore, the solutions of cluster “5” were discarded.

Noteworthy, unlike TLR1/TLR2, the TL4/MD-2 complex is symmetric (**Supplementary Figure 6D**). In that sense, we noticed that the binding modes of clusters “3” are the same but reflected along the symmetry axis (**Supplementary Figure 6D**). Thus, these two can be seen as the same cluster. The best binding mode of the cluster “3-6” showed contacts with epitopes “0”, “1”, “3” and “10”. It showed 3 hydrogen bonds and 2 salt bridges and had a binding energy of -9.9 kcal/mol (**Figure 9D**). The best binding mode of the cluster “1” showed contacts with epitopes “1”, “5”, “8 and “9” (**Supplementary Table 9**).

#### Simulating the immune response

**Figure 10:**
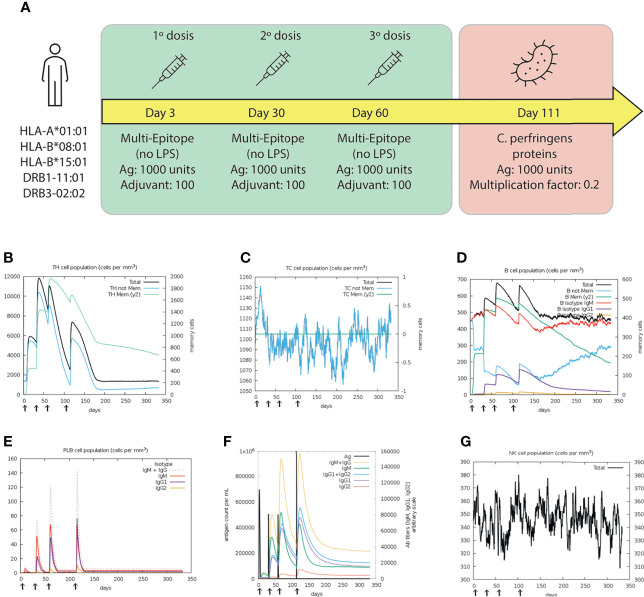
Results of the C-IMMSIM simulation for 350 days. **(A)** Schematic illustration of the vaccination trial, with three doses of the multi-epitope vaccine (green box) at days 3, 30, 60; and the challenge (red box) at day 111. Dynamics of **(B)** HTL and **(C)** CTL populations. Memory and not memory cells are represented with light-blue and green lines, respectively. **(D)** B cell populations, grouped by immunoglobulin isotype production. **(E)** Population of Plasma B lymphocytes producing IgM, IgG1 and IgG2. **(F)** Antigen concentration and relative antibodies responses. **(G)** Total population of NK cells. The first, second and third doses were inoculated.

The simulation of immunizing with the multi-epitope protein showed that the second and third doses generated significantly higher responses than the first one, as expected. HTL populations were higher in the second and third dose than in the first one, suggesting activation of the memory cells (**Figure 10B**). However, the CTL population was higher during the first dose, indicating an early immune response (**Figure 10C**). The B-cell subpopulations, including memory B-cells and Plasma B lymphocytes (PLB cells), showed considerable expansion after each dose reaching the highest peak at day 60 (**Figure 10D**). After the challenge, the response generated by B-cells was the highest in the simulation, indicating an appropriate production of antibodies (**Figure 10E**). Moreover, an early production of IgM was detected, which changed to IgG after the antigen administration (fourth response) (**Figure 10F**). Regarding the innate system, NK (natural killer) activity was found to be constant during the three doses and showed increased activity during the challenge (**Figure 10G**).

#### Codon optimization of MEP_12 DNA sequence

The optimized sequence of the 450 nt cDNA vaccine construct had a Codon Adaptation Index (CAI) of 0.772, and a %GC of 48.9%. This sequence contains 111 restriction sites for 55 commercial enzymes (**Supplementary Table 10**).

## Discussion

*C. perfringens* is one of the most common food-poisoning causing bacteria, causing a major impact on human health worldwide. It is also a reported biological warfare agent. These reasons make developing a vaccine an urgent matter. Most experimental studies have focused on *C. perfringens* toxins to find vaccines protective against gas gangrene. It has been shown that the alpha toxin protect against *C. perfringens* type A (67, 68). This toxin has been produced in endospores of recombinant *Bacillus subtilis* and tested as vaccine in mice, resulting in protection against gas gangrene (69). The *β*-toxin has also been evaluated as a possible vaccine against *C. perfringens* type C in piglets (70). However, none of them have been tested in humans. There is still no approved vaccine for humans nowadays (5).

While other studies had focused only on specific proteins of *C. perfringens* (71, 72), we consider that analyzing the whole proteome could reveal novel more immunogenic proteins. We followed three approaches to propose vaccine candidates: a whole-protein based vaccine, a protein-subunit based vaccine, and a multi-epitope protein vaccine. The first approach consist in identifying natural proteins of the pathogen enriched with both CTL and HTL epitopes to be used as recombinant vaccines. With the second approach, we further studied the sequence and structure of the main toxins, identifying regions exposed to the extra-cellular medium and rich in HTL epitopes that could generate immune responses against gas gangrene. Lastly, in the third approach, we identified the best CTL and HTL epitopes of the whole proteome and performed several immunoinformatics and structural analyses to assemble them into a multi-epitope construct, aiming to combat *C. perfringens* infection. The immunogenic proteins obtained by computational methods in these three approaches may represent good vaccine candidates, and they could be used as well for the development of immunodiagnostic tests (73, 74).

The immunoinformatic exploration of the whole proteome resulted in 429809 CTL- and 121450 HTL- predicted epitopes (**Supplementary Table 3**), which could be further used in both computational and experimental studies seeking a better understanding of the immunogenic characteristics of *C. perfringens* proteins, the developing of diagnostic tests, and the design of peptide-based vaccines and therapies. A strong positive correlation between the number of CTL- and HTL-epitopes predicted with the protein length was found, indicating that longer proteins contain a higher number of epitopes. This has been previously reported by an independent study in viral proteins, showing that protein length is positively correlated with its number of CD8+ T-cell epitopes within (75), supporting our observation. Another observation is that the 9-mer CTL-epitopes were more promiscuous, binding more HLA-I supertype alleles (**Supplementary Figure 1B**). These two observations might be correlated with the fact that most of the experimental data, and consequently most software training data, is based on 9-mer CTL-epitopes. A similar pattern was observed for 15 aa HTL-epitopes. These observations match with was is biologically expected, as they are the canonical epitope sizes which have been commonly observed in MHC-epitope experimental data (29, 76, 77).

The Collagenase A (UniProt ID: P43153), Exo-alpha-sialidase (UniProt ID: Q8XMY5), alpha n-acetylglucosaminidase (UniProt ID: Q8XM24) and hyaluronoglucosaminidase (UniProt ID: P26831) were identified as the top 4 proteins with more CTL- and HTL-epitopes. The Collagenase A, encoded by the gene colA, is an extracellular proteolytic enzyme that degrades extracellular matrix and plays a role in the pathogenesis of gangrene (78). This enzyme has hemorrhagic and dermonecrotic activities, and intravenous inoculation of Collagenase A has shown to be lethal for mice (79). However, little is known about its role as immunogen. The Exo-alpha-sialidase, encoded by the gene nanJ, is the largest of the three sialidases produced by *C. perfringens*. It is involved in the intestinal virulence by increasing the binding affinity of the sialidases to their targets, enhancing pathogen adherence to intestinal cells. Sialidase inhibitors, such as Siastatin B or N-acetyl-2,3-dehydro-2-deoxyneuraminic acid (NADNA), have been tested *in vitro* and proposed as possible therapeutics against intestinal infection of *C. perfringens*; however, *in vivo* validation is still needed (80, 81). The hyaluronoglucosaminidase, encoded by the gene nagH, is a carbohydrate-active enzyme that acts on the connective tissue during the gas gangrene (82, 83). The alpha n-acetylglucosaminidase is an enzyme with strong preferences for carbohydrate motifs, found on the class III mucins within the gastric mucosa (81). Although there is no study testing alpha-acetylglucosaminidase, vaccination with beta-acetylglucosaminidase has shown to generate protection against necrotic enteritis in chickens (84). Similarly, a peptide-based vaccine comprising of several epitopes from these mucolytic enzymes produced reduction of lesions caused by necrotic enteritis in chicks. Altogether, these studies suggest that these four enzymes can be used as potential protein-based vaccines (85). The use of epitope-enriched proteins has been previously suggested as a strategy for the rational selection of immunogens, considering B-cell epitopes (86) and T-cell epitopes (73). Proteins with high epitope density are expected to maximize the probability of immune cells presentation and activation. Protein-based vaccines have been widely studied and used, with some advantages like having relatively low production costs, and not causing severe side effects unlike attenuated vaccines (87, 88).

Next, we studied the six most studied *C. perfringens* type A toxins in more detail. All of them, except for the beta2 toxin, showed at least 10 HTL-epitopes by HLA-II supertype allele (**Figure 2C**). These observations suggest that toxins are generally good candidates for protein-based vaccines, as shown in previous experimental studies (89–93). Our results support the idea that toxins could generate an appropriate humoral response to protect, mainly, against gangrene or histotoxic damage before infection (67, 94). Noteworthy, we show the relevance of not only predicting promiscuous epitopes but studying their location within the proteins as well. For instance, we found that the most promiscuous epitopes of enterotoxin D are in SH3B domains, which are correlated with promoting pathogen survival and invasion by binding to host receptors. This indicates that not all the extension, but specific regions of the protein are rich in epitopes. The use of non-toxic domains of toxins has shown to be advantageous for vaccine development as they present similar immunogenicity than the entire toxins without undesired toxicity (95–97). For example, the use of the non-toxic domain HC50 of the Botulinum neurotoxin type A induces a strong anti-HC50 IgG antibody response, neutralizing the circulating neurotoxin in mice (98). And, immunization with the non-toxic fragment of the C-domain of phospholipase C produces antibodies against this toxin, providing protection against gas gangrene in mice (5). Similarly, our analysis suggests that the non-toxic domain of Perfringolysin O is a promissory candidate for experimental testing as a protein-subunit based vaccine.

Numerous studies have used bioinformatics software to propose epitope-based vaccines against *C. perfringens*. A previous study predicted B-cell epitopes in the epsilon toxin of *C. perfringens* types B and D. However, they are not the main toxinotypes of *C. perfringens* affecting human health (99) and the epsilon toxin is not present in *C. perfringens* type A. Another study predicted 15 unique epitopes in the toxin NetF using mouse rather than human MHC alleles. Although the authors suggest that NetF could be a good vaccine candidate and the epitopes found can be used in multi-epitope vaccines, this needs to be evaluated in humans (100). Furthermore, the NetF protein has been associated with gastroenteritis and enterocolitis in canine and foals (100), but its role in humans is not well studied. Another study has predicted T- and B-cell epitopes in the fructose 1,6-bisphosphate aldolase (101). Nonetheless, they did not design a multi-epitope construct, which might be more effective than single-epitope vaccines (18, 102). Other bioinformatic studies of *C. perfringens* aimed to find only candidate epitopes in toxins or few proteins (71, 103). However, exploring the whole proteome, as done in the present study, allows the computational identification of a broader set of potential epitopes that may trigger better immune responses, as seen in other pathogens like SARS-CoV-2 (104).

A rational workflow was elaborated to design a multi-epitope vaccine, consisting of (1) using different immunoinformatics tools to predict epitopes from the whole proteome of *C. perfringens* (2), considering the epitope location in the structure of their native proteins, and (3) merging and assembling nested epitopes in potential constructs, and (4) evaluating the structure and dynamics of the constructs by MD. Of the programs available for T-cell epitope prediction, we opted to use NetMHCpan and MHCFlurry to predict CTL-epitopes, and NetMHCIIpan for HTL-epitopes, as they have shown the best performance against competitors (27, 28). Proteasome cleavage and TAP transport are also important processes of the intracellular presentation pathway. Predictors of proteasome developed, being the most popular netChop and ProteaSMM. However, benchmarking studies have shown that these predictors still need to improve their sensitivity and specificity (105). Moreover, it has been reported that current cleavage predictions based on *in vitro* data do not correlate with *in vivo* data (106). Therefore, we decided not to discard constructs based on these predictors. TAP transport predictors face the same limitations, with very few predictors available and the lack of unbiased benchmarking studies (107).

Targeting HLA supertype alleles maximize the potential usability of our multiepitope in different populations. This choice was a tradeoff between wider usability and higher local specificity, and we decided to prioritize the first one as currently *C. perfringens* infection is a widely spread pathogen without universal vaccine. Higher local specificity would be more relevant in other scenarios, such as if broad-range vaccines become available, or if the disease becomes endemic, or if there is a high incidence of cases in a specific geographic location, among others. This will imply a change in the HLA allele selection strategy, giving more importance to certain HLA alleles abundant in the specific geographic areas affected by the disease.

Keeping the synthetic protein small is important to reduce synthesis and production costs (i.e. facilitate its purification in inclusion bodies), as well as to prevent toxicity in the organism used for production (98, 108). Using nested epitopes allows to maximize the number of epitopes in the multi-epitope construct without increasing the construct length. Recent studies have used nested epitopes to construct multi-epitope proteins, but experimental testing is still needed to verify the advantage provided by this strategy (104, 109). From the set of 112714 nested epitopes, several filters were applied to select the best candidates. One of these filters is to prioritize epitope promiscuity, which has been associated with contributing to epitope immunodominance, as promiscuous epitopes are recognized by multiple HLA alleles (110). The use of promiscuous epitopes allows to cover a larger number of HLA-alleles (i.e. a larger proportion of the target population) without increasing the number of epitopes in the vaccine construct (111). We also filtered by epitope conservation among all the reported protein variants of *C. perfringens*. This may result in covering a broader range of current, and potentially future, pathogen variants. Thus, a high epitope conservation could lead to a better protection as the immune response tends to focus on conserved epitopes when individuals are exposed to different strains (112, 113). In our construct, the conservation analysis of the overlapped nested epitopes showed 100% conservation. Thus, even though *C. perfringens* type A is the most common cause of gas gangrene, our construct may confer extended protection against other toxinotypes as well. Our construct of 150 aa is made of 9 overlapped HTL-CTL epitopes, comprising 24 HTL-epitopes containing 34 CTL-epitopes. This suggests a better cellular immune response than previous multiepitope constructs, which were longer and had less epitopes in its sequence (114). Whilst the study of Aldakheel *et al.* (114) attempted to target all HLA-I and -II alleles, we opted to focus on the HLA supertype alleles. This allowed us to need less epitopes in our design to match all the target alleles. Additionally, having less HLAs to target allows epitopes with better immunogenicity to be selected from the prediction. Thus, we successfully covered all the HLA supertype alleles, while the previous study just covered 10 of the 12 HLA-I and 5 of the 8 HLA-II supertypes alleles. Additionally, our design is about 1/3 in length (150 versus 415 aa), due to our strategy of using nested HTL-CTL epitopes in extracellularly exposed regions.

The 9 overlapped nested epitopes in our construct belong to the FTsX domain-containing protein (UniProt ID: Q8XM39), the cardiolipin synthase (UniProt ID: P0C2E2), the Polysaccharide synthase 2 domain-containing protein (UniProt ID: Q8XN75), the probable hemolysin-related protein (UniProt ID: Q8XPD3), the TraG-D C domain-containing protein (UniProt ID: Q93M96), the spore germination protein KA (UniProt ID: Q8XMP0) and CPE0011 (UniProt ID: Q8XPF2). Our prediction indicates that all of them are membrane proteins with a high content of HTL- and CTL-epitopes. Moreover, these proteins present functions related with the cell membrane structure (Cardiolipin synthase), survival (Spore germination protein KA) and host colonization (Hemolysin-related protein) (115–117). External regions of transmembrane proteins involved in the infective process are frequently considered vaccine targets, as antibodies can efficiently neutralize them (118).

Assembling epitopes to make a new protein can be performed with or without linkers, or adding adjuvant sequences (119). Multiple linker and adjuvant sequences have been reported in the literature (120–122). Linkers are used to reduce the occurrence of neoepitopes (123), with the downside of increasing the construct length and the cost of protein synthesis. There are very few software to optimize the use of linkers (112, 124, 125), and this approach is frequently used because finding an appropriate epitope sorting without linkers is time-consuming and computationally demanding. Here, we used a novel algorithm and software based on graph theory (EpiSorter), assembling all the epitopes without the need of linkers and without unwanted neoepitopes shared with human proteins, avoiding autoimmune or tolerance responses. Thus, we believe that using linkers can be avoided and, instead, we recommend prioritizing the number and promiscuity of the epitopes selected.

Several immunoinformatics studies have reported structural models of their multi-epitope constructs using only one or two software, complemented with refinement steps (126–128). Nonetheless, modeling novel proteins is a difficult task as they usually do not have close homologues. The situation becomes even more complex if the novel protein to model consists of highly flexible linear epitopes. To tackle this problem, we employed the state-of-the-art protein-structure prediction software AlphaFold2 (47). Nowadays, this is the first time this software has been used to predict the 3D structure of multi-epitope constructs.

The high proportion of residues of the multi-epitope structures with low pLDDT suggests that the models obtained by AlphaFold were not completely reliable. However, it also says that the multi-epitope protein is highly disordered and flexible, characteristics that may favor its binding to immune proteins. Disordered regions in proteins are characterized by a lack of a stable tertiary structure and high flexibility (129). Moreover, it has been pinpointed that flexibility in protein antigens positively affects their binding affinity (130).

The multi-epitope construct selected did not present Ramachandran outliers and shows an ERRAT score comparable to scores of high-resolution structures (131). Moreover, these quality statistics are better than the ones obtained in various similar studies designing multi-epitope vaccines (119, 127, 132–135). This reaffirms the quality and consistency of our structural approach, although better modeling software is still needed for multi-epitope proteins.

Docking was performed against TLR1/TLR2 and TLR4/MD2, as they have been widely reported to be important in the innate defense against *C. perfringens* infection in chickens and mice (136), although little is known in humans. The lipopeptide and lipopolysaccharide binding sites of TLR1/TLR2 and TLR4/MD-2, respectively, have been structurally characterized (137, 138). As there is neither prior experimental information about the binding sites for MEPs, nor which epitope of our novel MEP might interact with the TLRs, we considered blind docking the most appropriate approach. The docking simulation showed binding between our MEP_12 and the TLR1/TLR2, suggesting an innate immune response in addition to the adaptive immune response generated by the nested epitopes. The docking analyses also showed that epitopes “3” and “10” interact with TLR1/TLR2. These epitopes are relatively rigid, which may result in a more favorable binding as shown in previous studies. Epitopes tend to be more rigid than the rest of the protein (31). And, from a thermodynamic point of view, rigid surfaces have less entropic penalty when interacting with other proteins, resulting in tighter bindings.

The binding region of MEP_12 in TLR4/MD-2 is similar to the one observed in a previous study, where a SARS-CoV-2 candidate vaccine was docked against this receptor (119), even though different protocols were followed. This suggest that this region of TLR4/MD-2 is where antigenic proteins bind. However, experimental studies are needed to validate this observation. Noteworthy, the MEP_12 is smaller and establishes fewer interactions with TLR4/MD-2 than the SARS-CoV-2 candidate vaccine but achieves a similar binding energy, differing in just in 0.4 kcal/mol. This indicates that our design is a more efficient TLR4/MD-2 binder and correlates with the finding that MEP_12 is flexible and disordered. These are two desired characteristics, as they make antigens more efficient at binding immune proteins (33).

Immunization with MEP_12 was simulated, showing that our vaccine candidate can elicit immune responses to clear the antigen on secondary exposure. The challenge with *C. perfringens* proteins after three vaccine injections induced higher levels of IgG than IgM. IgM is the principal isotype in the first response, while IgG is predominant in secondary responses, representing specific pathogen recognition (139). Also, the increased production of immunoglobulins by plasma B cells indicated that memory of *C. perfringens* proteins is present in the immune system (140). Finally, a gene encoding MEP_12 was designed optimizing its sequence for expression and production in *E. coli*, as this organism has been widely used to produce recombinant vaccines (141–143). Additionally, the design can be adapted to other organisms following the methodology we described.

In summary, in the current study, we have performed a thorough immunoinformatic exploration of the whole proteome to generate vaccine candidates against *C. perfringens*. Three approaches were followed to identify (1) the most immunogenic proteins (2), immunogenic non-toxin domains of toxins, and (3) the design a novel protein with the best HTL-CTL nested epitopes, expected to trigger both adaptive and cellular immune responses. These resulted in promising candidates for further experimental *in vitro* and *in vivo* studies. These candidates may help in the prevention of necrotic enteritis, as well as other human diseases caused by *C. perfringens*.

## Data availability statement

The original contributions presented in the study are included in the article/**Supplementary Material**. Further inquiries can be directed to the corresponding author.

## Author contributions

Conceptualization, LFS and DR. Methodology, LFS, DR, GJ-A, and DR. Software, LFS, DR, and GJ-A. Validation, LS and DR. Formal Analysis, LFS and DR. Investigation, LFS, AR, GJ-A, and DR. Resources, DR. Data curation, DR. Writing – original draft preparation, LFS, DR, GJ-A, and DR. Writing-review and editing, LFS, AR, and DR. Visualization, LFS and GJ-A. Supervision, DR. Project administration, DR, RLL, YS, and CO-R. Funding acquisition, DR, RLL, YS, and CO-R. All authors contributed to the article and approved the submitted version.

## Funding

The publication of this work was funded by the grant N°2264962 SNIP N°317435 “Creación del Centro de Promoción de la Investigación y Transferencia Tecnológica” of the University “Toribio Rodriguez de Mendoza de Amazonas”.

## Conflict of interest

The authors declare that the research was conducted in the absence of any commercial or financial relationships that could be construed as a potential conflict of interest.

## Publisher’s note

All claims expressed in this article are solely those of the authors and do not necessarily represent those of their affiliated organizations, or those of the publisher, the editors and the reviewers. Any product that may be evaluated in this article, or claim that may be made by its manufacturer, is not guaranteed or endorsed by the publisher.

## Supplementary material

Supplementary Figure 1 Density and promiscuity of the CTL- and HTL-epitopes. **(A)** Epitope density in proteins by epitope length (CTL-epitopes: 8, 9 and 10 aa, HTL-epitopes: 15 aa). **(B)** The bar plot represents in the X-axis the number of epitopes identified, for each epitope length (Y-axis). The color indicates how many HLA supertype alleles each epitope binds. In the bar plots **(C)** (for CTL-epitopes) and **D** (for HTL-epitopes), the Y-axis represents the number of epitopes that can bind a certain number of HLA supertype alleles (X-axis). Panels **(E, F)** show the distribution of the number and density, respectively, of HTL-epitopes in the proteins of each of the levels of evidence indicated in the X-axis.

Supplementary Figure 2 Conservation of the nested candidate epitopes across C. perfringens toxinotypes. Each subfigure shows the alignment of the nested epitope, the epitope ID and the protein name.

Supplementary Figure 3
**(A)** Mean pLDDT of the 22 multi-epitope (MEP) candidates. **(B)** Structural alignment between the centroid of the most populated cluster of MEP_12, before (orange) and after (cyan) refinement.

Supplementary Figure 4 Quality assessment of the centroid of the most populated cluster of MEP_12 before refinement. **(A)** Ramachandran plot for the general case. The percentage of residues in preferred (light blue) and allowed (blue) regions are shown. **(B)** Scatterplot of the Z-scores of the centroid (black dot) and protein structures with experimental evidence (NMR: blue, X-ray crystallography: light blue). **(C)** ERRAT plot of the centroid. Bars represent the error value (white: error < 95%, yellow: 95% error < 99%) of a nine-residue sliding window. The overall quality factor indicates the percentage of protein residues with error values lower than 95%.

Supplementary Figure 5 SASA values per epitope. Values over the last 500 ns of convergent simulation are shown for epitopes “0” to “10” (excluding “4”) in panels **A** to **I**, respectively. The SASA of all epitopes shown but “5” and “9” achieved convergence. Epitope “8” only achieved convergence during the last 400 ns (enclosed in a red box, close-up in inset plot). So, only this region was considered for its mean SASA computations.

Supplementary Figure 6 Comparison of all plausible clusters of docking of MEP_12 against TLR1/TLR2 and TLR4/MD-2. The best binding modes of clusters having Haddock-scores with overlapping error bars (standard deviations) are shown for the dockings **(A)** MEP_12-TLR1/TLR2, and **(C)** MEP_12-TLR4/MD2. TLR1 and TLR2 chains are colored in green and orange, respectively. Attached glycans are shown as sticks in cyan. In **(A)**, the best binding modes of MEP_12 from clusters “1”, “2”, “3”, and “4” are colored in red, cyan, gray, and blue, respectively. In **(C)**, the best binding modes of MEP_12 from clusters “1”, “3”, “5”, and “6” are colored in gray, blue, red, and cyan, respectively. **(B)** Enlarged view of the best binding mode of cluster “1” (red cartoon with transparent surface) in the docking MEP_12-TLR1/TLR2. The N-terminal residues missing in TLR1/TLR2 structure (in gray) were completed by structural alignment to a TLR2 model from the AlphaFold Protein 1300 Database. These interact with MEP_12 cluster “1”. **(D)** Best binding modes from cluster “3” and “6” from the docking MEP_12-TLR4/MD2. The black line separating the two sides of TLR4/MD-2 represents a symmetry axis. The binding modes of left side become equivalent to the right side after a 180° right-handed rotation, as shown in **(E)**.
