# Supplementary figures and images for: Immunoinformatic analysis of the whole proteome for vaccine design: An application to Clostridium perfringens
Source: Front Immunol. 2022 Aug 30;13:942907. doi: 10.3389/fimmu.2022.942907 (PMC9469472; doi:10.3389/fimmu.2022.942907)

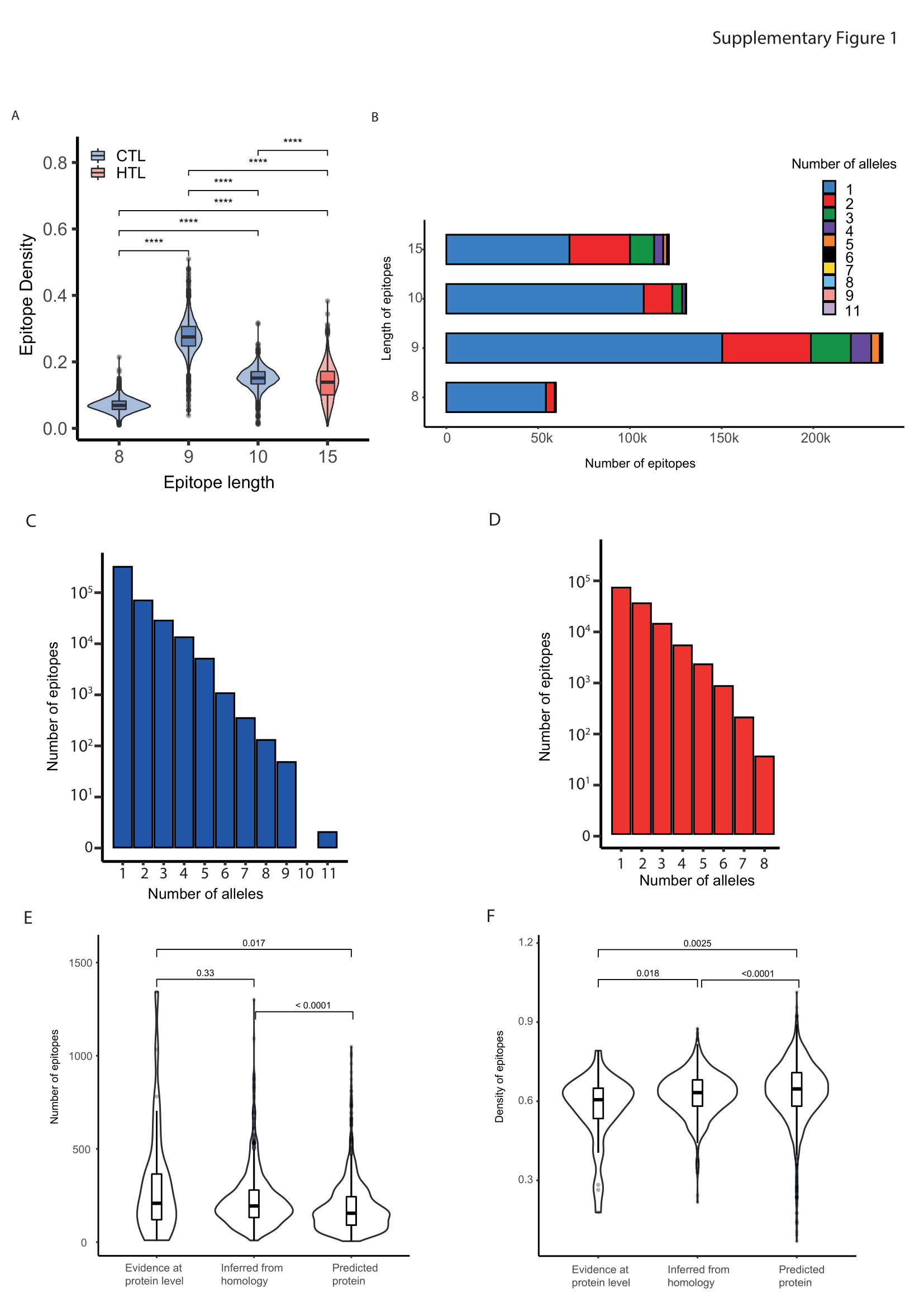

Supplement: Supplementary Figure 1 — Density and promiscuity of the CTL- and HTL-epitopes. (A) Epitope density in proteins by epitope length (CTL-epitopes: 8, 9 and 10 aa, HTL-epitopes: 15 aa). (B) The bar plot represents in the X-axis the number of epitopes identified, for each epitope length (Y-axis). The color indicates how many HLA supertype alleles each epitope binds. In the bar plots (C) (for CTL-epitopes) and D (for HTL-epitopes), the Y-axis represents the number of epitopes that can bind a certain number of HLA supertype alleles (X-axis). Panels (E, F) show the distribution of the number and density, respectively, of HTL-epitopes in the proteins of each of the levels of evidence indicated in the X-axis. [file Image_1.tiff]

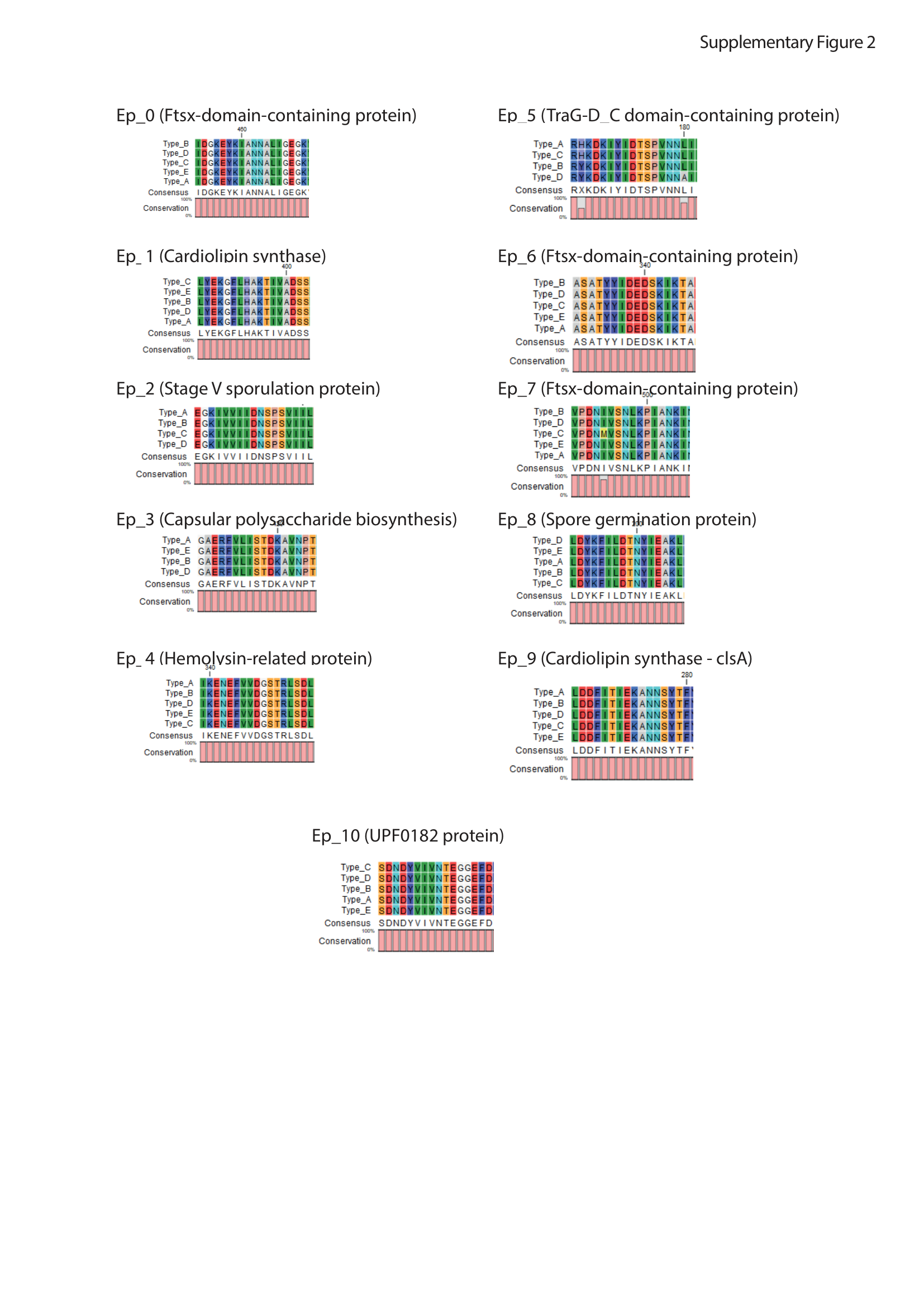

Supplement: Supplementary Figure 2 — Conservation of the nested candidate epitopes across C. perfringens toxinotypes. Each subfigure shows the alignment of the nested epitope, the epitope ID and the protein name. [file Image_2.tiff]

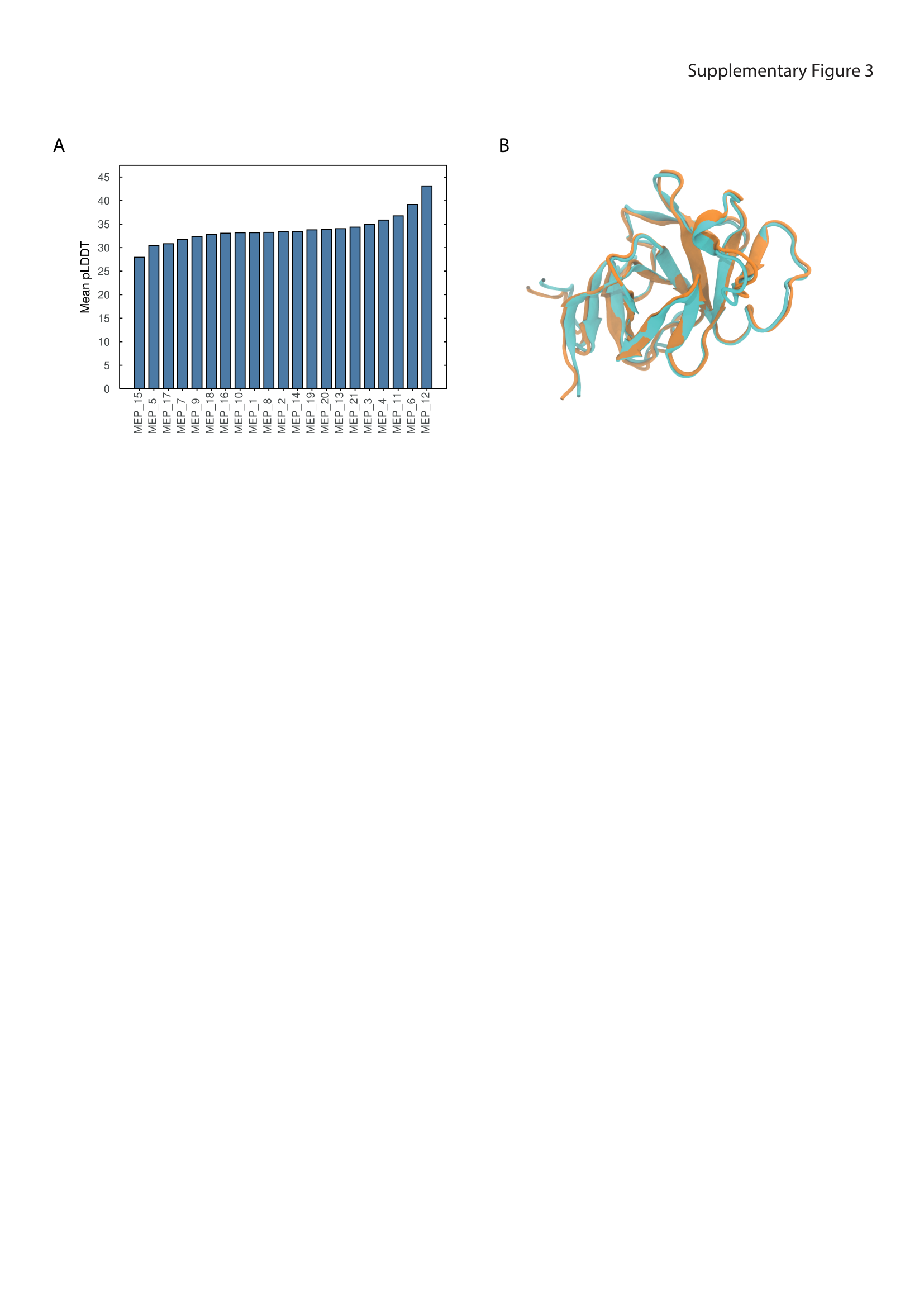

Supplement: Supplementary Figure 3 — (A) Mean pLDDT of the 22 multi-epitope (MEP) candidates. (B) Structural alignment between the centroid of the most populated cluster of MEP_12, before (orange) and after (cyan) refinement. [file Image_3.tiff]

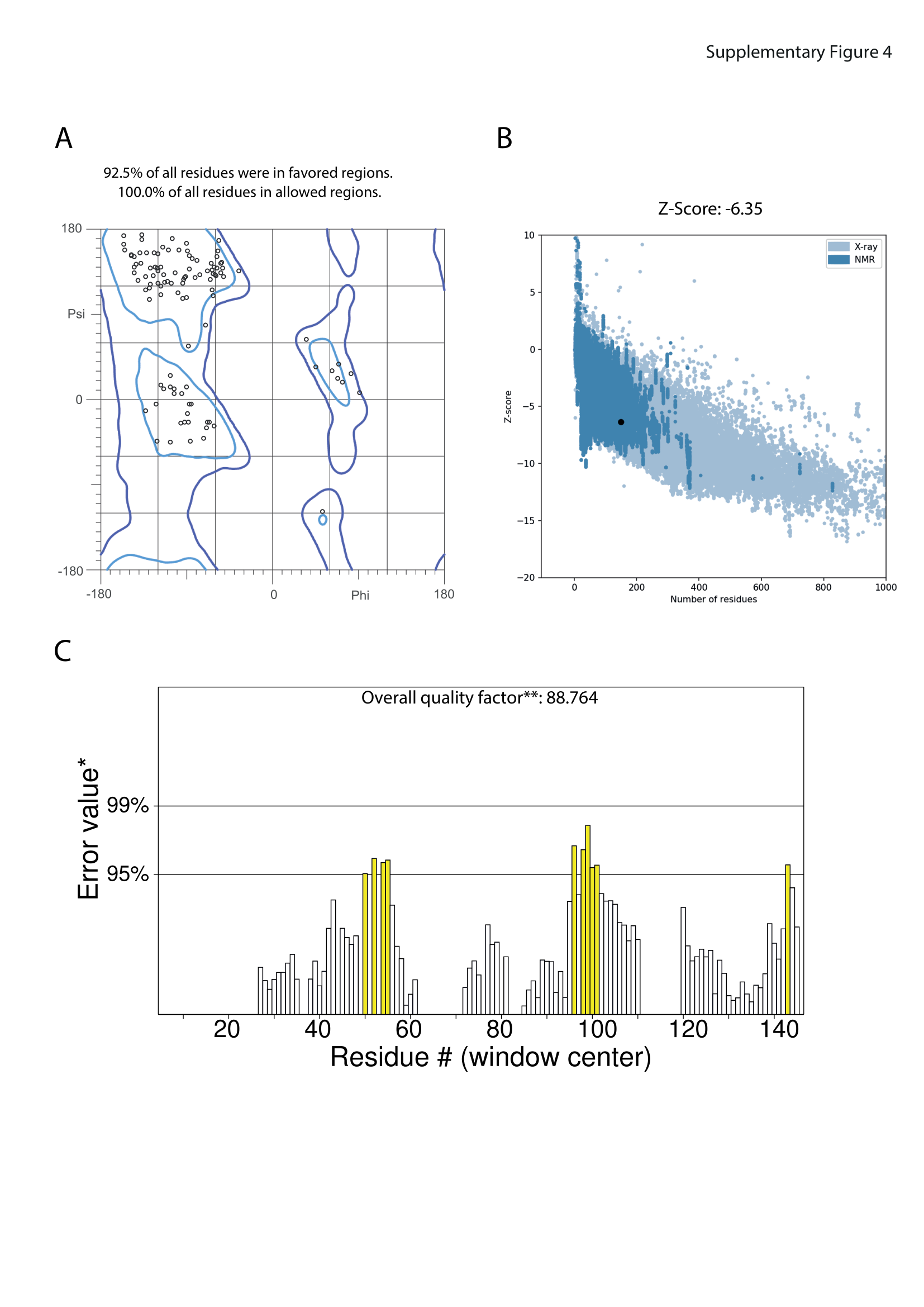

Supplement: Supplementary Figure 4 — Quality assessment of the centroid of the most populated cluster of MEP_12 before refinement. (A) Ramachandran plot for the general case. The percentage of residues in preferred (light blue) and allowed (blue) regions are shown. (B) Scatterplot of the Z-scores of the centroid (black dot) and protein structures with experimental evidence (NMR: blue, X-ray crystallography: light blue). (C) ERRAT plot of the centroid. Bars represent the error value (white: error < 95%, yellow: 95% error < 99%) of a nine-residue sliding window. The overall quality factor indicates the percentage of protein residues with error values lower than 95%. [file Image_4.tiff]

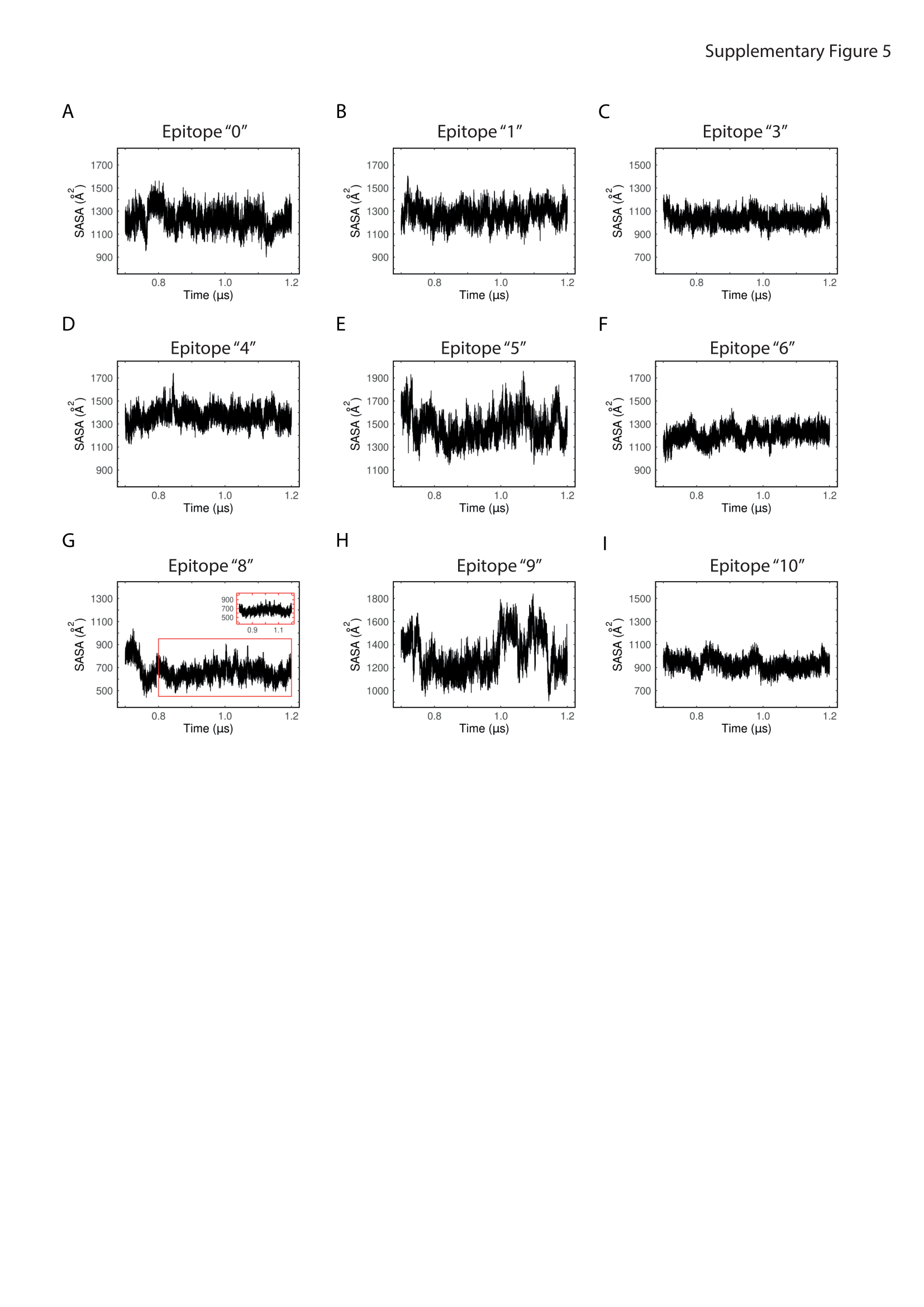

Supplement: Supplementary Figure 5 — SASA values per epitope. Values over the last 500 ns of convergent simulation are shown for epitopes “0” to “10” (excluding “4”) in panels A to I, respectively. The SASA of all epitopes shown but “5” and “9” achieved convergence. Epitope “8” only achieved convergence during the last 400 ns (enclosed in a red box, close-up in inset plot). So, only this region was considered for its mean SASA computations. [file Image_5.tiff]

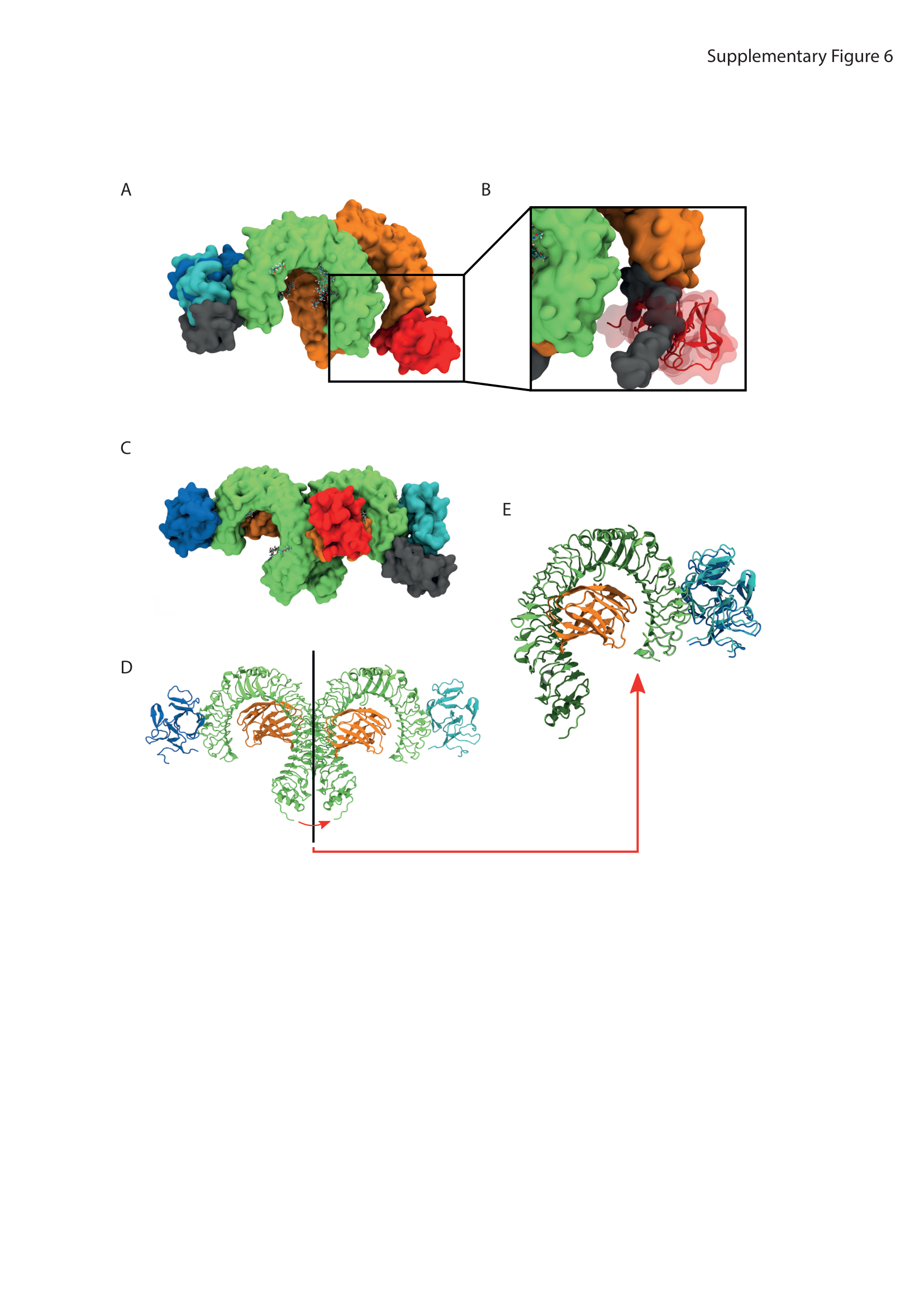

Supplement: Supplementary Figure 6 — Comparison of all plausible clusters of docking of MEP_12 against TLR1/TLR2 and TLR4/MD-2. The best binding modes of clusters having Haddock-scores with overlapping error bars (standard deviations) are shown for the dockings (A) MEP_12-TLR1/TLR2, and (C) MEP_12-TLR4/MD2. TLR1 and TLR2 chains are colored in green and orange, respectively. Attached glycans are shown as sticks in cyan. In (A), the best binding modes of MEP_12 from clusters “1”, “2”, “3”, and “4” are colored in red, cyan, gray, and blue, respectively. In (C), the best binding modes of MEP_12 from clusters “1”, “3”, “5”, and “6” are colored in gray, blue, red, and cyan, respectively. (B) Enlarged view of the best binding mode of cluster “1” (red cartoon with transparent surface) in the docking MEP_12-TLR1/TLR2. The N-terminal residues missing in TLR1/TLR2 structure (in gray) were completed by structural alignment to a TLR2 model from the AlphaFold Protein 1300 Database. These interact with MEP_12 cluster “1”. (D) Best binding modes from cluster “3” and “6” from the docking MEP_12-TLR4/MD2. The black line separating the two sides of TLR4/MD-2 represents a symmetry axis. The binding modes of left side become equivalent to the right side after a 180° right-handed rotation, as shown in (E). [file Image_6.tiff]
